# Optimal control and cost-effectiveness analysis of PfSPZ vaccination and indoor residual spraying in malaria transmission: a case study in Keerom

**DOI:** 10.3389/fpubh.2026.1872970

**Published:** 2026-07-01

**Authors:** Iffatricia Haura Febiriana, Dipo Aldila, Puji Budi Setia Asih, Ismail Ekoprayitno Rozi, Lervianna Sitanggang, Abdul Gani Raflythenu, Lepa Syahrani, Muhamad Hifzhudin Noor Aziz, Olumuyiwa James Peter, Putri Zahra Kamalia

**Affiliations:** 1 Department of Mathematics, Faculty of Mathematics and Natural Sciences, Universitas Indonesia, Depok, Indonesia; 2 Eijkman Research Center for Molecular Biology, National Research and Innovation Agency (BRIN), Cibinong, Indonesia; 3 Innovative Mathematics and Predictive Analytics for Complex System and Technology Laboratory (IMPACT Lab), Universitas Indonesia, Depok, Indonesia; 4 Keerom District Health Center, Papua, Indonesia; 5 Malaria Consultant (UNICEF), Jakarta, Indonesia; 6 Faculty of Science, Institute of Mathematical Sciences, Universiti Malaya, Kuala Lumpur, Malaysia; 7 Department of Mathematics, Saveetha School of Engineering, SIMATS, Saveetha University, Chennai, Tamil Nadu, India; 8 Department of Mathematical and Computer Sciences, University of Medical Sciences, Ondo, Nigeria

**Keywords:** cost-effectiveness, indoor residual spraying, Keerom, malaria, PfSPZ vaccine

## Abstract

This study aims to investigate the potential impact of the Plasmodium falciparum sporozoite (PfSPZ) vaccine and indoor residual spraying (IRS) on malaria transmission in Keerom, Papua. An optimal control model is introduced in this article for malaria transmission using a deterministic compartmental host–vector model. The model considers the potential impact of the new PfSPZ vaccine and IRS through a novel host–vector framework. Treatment failure is also accounted for in the model to provide a more realistic representation of the disease dynamics. Mathematical analysis regarding the existence and stability of equilibria is conducted rigorously, and the basic reproduction number is derived using the next-generation matrix approach. We found that the malaria-free equilibrium is always locally asymptotically stable when the basic reproduction number is less than 1. Conversely, using a center manifold approach, we showed that the malaria-endemic equilibrium is asymptotically stable when the basic reproduction number is greater than 1 but close to 1. Model parameter values are estimated using incidence data from Keerom, Papua, an area in Indonesia with one of the highest malaria incidences at the national scale. A global sensitivity analysis is conducted using Partial Rank Correlation Coefficients, which reveal the importance of vaccination and IRS strategies in reducing the basic reproduction number. Through cost-effectiveness analysis based on the optimal control simulation results, we find that although the IRS-only intervention appears to be the most cost-effective strategy, the combination of PfSPZ vaccination and IRS yields the greatest overall impact on reducing malaria transmission. In particular, the combined strategy produces the largest reduction in the number of infected individuals, while the IRS-only strategy gives the most favorable cost-effectiveness outcome. These results suggest that PfSPZ vaccination and IRS can provide important benefits for malaria control in high-endemic areas such as Keerom, Papua.

## 1 Introduction and model novelty

### 1.1 Recent facts on malaria

The world harbors a myriad of communicable diseases, ranging from those transmitted through direct human contact to those requiring vectors or environmental transmission. Diseases requiring a vector for transmission are also known as vector-borne diseases. Examples of such diseases include dengue fever, malaria, and chikungunya. Among vector-borne diseases, malaria has the highest annual incidence globally, especially in Africa and Asia (1). Malaria is prevalent in tropical regions worldwide, including Africa, Asia, and Latin America. Sub-Saharan Africa bears the highest burden of malaria cases and deaths (2).

Indonesia is one of the countries that contributes to a high number of vector-borne disease cases, with malaria remaining an important priority despite substantial progress toward elimination. In Indonesia, malaria transmission is highly heterogeneous and is concentrated mainly in the eastern region, particularly Papua. National malaria data also show that Papua contributes the majority of positive malaria cases in Indonesia, indicating that the success of malaria elimination in Indonesia strongly depends on malaria control in this province. In this context, Keerom Regency is an important study area because it is one of the malaria-endemic districts in Papua, with a very high Annual Parasite Incidence (API) far above the national average. Therefore, malaria in Keerom is not only a local public health problem, but also an important barrier to the national malaria elimination agenda. Studying Keerom is also relevant from a broader perspective, as malaria transmission is increasingly concentrated in high-risk areas where additional or combined intervention strategies may be required. Hence, Keerom provides a meaningful case study for assessing the impact of PfSPZ vaccination and indoor residual spraying (IRS) in a high-endemicsetting.

Malaria is a life-threatening disease caused by the Plasmodium parasite and transmitted to humans through the bite of infected female Anopheles mosquitoes (3). Symptoms typically include high fever, chills, headaches, sweating, fatigue, body aches, and nausea. If left untreated, malaria can progress to severe and potentially fatal complications (4). Malaria can be diagnosed through laboratory tests that detect parasites in the blood (5). Early diagnosis and prompt treatment are crucial to prevent severe illness and death. In addition to these measures, the World Health Organization (WHO) recommends the use of insecticide-treated bed nets and indoor residual spraying (IRS) in malaria control efforts (2). Furthermore, the WHO considers early diagnosis and prompt treatment, insecticide-treated bed nets, and IRS the three main pillars of malaria control.

Effective malaria prevention strategies include the use of insecticide-treated bed nets (2), indoor residual spraying to kill mosquitoes (2), and antimalarial drugs for preventive treatment in high-risk areas (6, 7). Artemisinin-based combination therapy (ACTs) is an effective treatment for uncomplicated malaria (8). However, the emergence of drug-resistant malaria parasite strains poses a significant challenge to malaria control efforts (9). Another popular intervention for mosquito-borne diseases is the use of mosquito repellents. Mosquito repellents are substances or products designed to prevent mosquitoes from biting humans. They created a barrier through emitting odors that repel mosquitoes, thereby reducing the risk of mosquito-borne diseases (10). Global efforts to combat malaria have resulted in significant progress in reducing disease transmission. Increased funding for access to diagnostic tests and treatment, distribution of bed nets, IRS, and research into new prevention and treatment methods are crucial for further progress in malaria control and elimination.

In addition to the aforementioned interventions, one malaria prevention method is vaccination. Vaccination involves administering biological products to individuals at risk of disease. By administering vaccines, the immune system is stimulated to develop immunity, thereby strengthening an individual's resistance to disease. The most extensively studied and commonly marketed malaria vaccine is RTS,S/AS01. The RTS,S/AS01 vaccine is one of the vaccine types recommended by the WHO. This vaccine can combat malaria with an effectiveness of 40–50% (11) and has been shown to significantly reduce malaria morbidity and mortality due to P. falciparum in children. This recommendation is based on ongoing trial results in Ghana, Kenya, and Malawi (12). However, in Indonesia, the RTS,S/AS01 vaccine has not been implemented to date. Vaccines do not fall within the three main pillars discussed earlier because the WHO-recommended vaccine (RTS,S/AS01) varies in effectiveness across regions. For instance, it is nearly 40-50% effective in Africa but becomes ineffective when administered to individuals in other regions such as Thailand. Additionally, the parasite causing malaria is much more complex than the viruses and bacteria for which we have vaccines. Hence, biologists tend to be pessimistic about the use of vaccines for malaria prevention. However, in the past 3 years, vaccine research has been revitalized by biologists (13, 14). To date, the efficacy of the PfSPZ vaccine in the Indonesian population remains unknown. Furthermore, not only biological research but also mathematical research is needed to support the results of biological research. Thus, research can be conducted in parallel, and when biological and mathematical vaccine research is complete, the results can be implemented immediately.

In addition to the RTS,S/AS01 vaccine, the PfSPZ Vaccine consists of Pf sporozoites (SPZ) weakened using radiation, aseptically purified, cryopreserved, expressing thousands of proteins, and inducing protective immunity against early-stage malaria clinically through antibodies and T cells (15–18). The PfSPZ Vaccine has been studied to combat malaria with an overall effectiveness of 78% (14). Benjamin Mordmüller et al. sought to identify immunization regimens that provide similar vaccine efficacy against CHMI with homologous and heterologous Pf strains for at least 9 weeks in malaria-susceptible adults. Overall, VE was 78% (95% predictive interval: 57–92%), and against heterologous and homologous, it was 79% (95% predictive interval: 54–95%) and 77% (95% predictive interval: 50–95%), respectively. The PfSPZ Vaccine is safe and well-tolerated (14).

### 1.2 Existing mathematical model on malaria transmission

Many researchers have used mathematical models to understand how diseases can spread among populations; see Handari et al. (19), Tasman et al. (20), Aldila et al. (21, 22), Febiriana et al. (23, 24), and Chukwu et al. (25) for some examples. Numerous mathematical models have been developed to elucidate the mechanism of malaria transmission, beginning with Ross (26), followed by Macdonald (27), and later by other researchers. For example, Mojeeb conducted a study on mathematical models of malaria that account for vector-bias effects, concluding that malaria could worsen if control strategies are not improved (28). Ndii and Adi (29) developed a mathematical model of malaria that accounts for human awareness effects. In this model, the susceptible population is divided into unaware and aware individuals. However, the model needs to account better for the variability in individual awareness levels. Ndii recommends enhancing human awareness programs to reduce malaria transmission rates and case numbers (29). Olaniyi et al. (30) discussed optimal control in a mathematical model of malaria, with indoor insecticide use as one of the control options. The findings indicate that vector control through indoor insecticide use alone is insufficient for malaria control. Additional interventions, such as bed net use or treatment, are necessary.

Recently, the authors in Febiriana et al. (23) introduced a malaria model that incorporates population awareness and the use of bed nets as control measures. Their cost-effectiveness analysis revealed that integrating media campaigns with bed net use significantly reduced malaria transmission. Researchers (20) discussed a more advanced model of malaria transmission, examining the impact of relapse, reinfection, and recrudescence. Their model highlighted intriguing bifurcating phenomena at a reproduction number of 1, driven by the treatment saturation parameter. Notably, a combination of backward, forward, and forward bifurcation with hysteresis was observed in their findings. In Handari et al. (19), the authors developed a mathematical model of malaria to investigate the impact of new vaccines and transmission-blocking drugs. Their sensitivity analysis demonstrated that increasing the mosquito mortality rate could effectively reduce the reproduction number, thereby curbing malaria's spread in the population. Additionally, the authors proposed an age-structured model that accounts for migration and immunity in Seck et al. (31). They demonstrated that malaria could be eliminated if the reproduction number remained below 1, whereas it would persist if it exceeded 1. Since the emergence of COVID-19 in 2019, several studies have also explored the co-infection dynamics of malaria and COVID-19, including those presented in Tchoumi et al. (32), Abioye al. (33), and Avusuglo et al. (34). These studies provide valuable insights into the interplay between these two significant diseases.

While numerous mathematical models have been developed to study malaria transmission, incorporating vaccination strategies, especially PfSPZ, remains a relatively unexplored area. Arafa et al. (35) introduced a mathematical model that accounts for both the proportion of vaccinated individuals and the vaccine's effectiveness. Their numerical simulations provided valuable insights, though the study did not specifically emphasize evaluating the vaccine's broader impact on malaria transmission. Nirwani et al. (36) developed a model where susceptible individuals were categorized as unvaccinated, and vaccinated individuals transitioned directly to the recovered compartment. Their studies contributed significantly to analytical understanding, with numerical results supporting this foundation. Affandi (37) explored optimal control by using vaccination as a strategy to reduce the transition rate from susceptible to latent individuals. The study successfully identified the optimal control framework for vaccination's influence, setting the stage for future simulations to further quantify its impact on malaria. Aldila (38) presented an innovative model integrating Tafenoquine treatment to prevent relapse in humans and fumigation to manage mosquito populations. This study highlighted that increasing the vector bias parameter can simultaneously increase the number of infected humans and reduce mosquito infections, underscoring the complexity of the relationship. Furthermore, the research emphasized the importance of well-timed fumigation for effective malaria control.

### 1.3 Our novelties

From the mathematical models for malaria discussed in the previous section, several important features of malaria dynamics remain underexplored. The first is the infection stages of individuals with malaria. Malaria infection is not always symptomatic; in many cases, the parasite remains dormant in the human liver. This dormancy makes it challenging to monitor their presence. The second feature to consider is the implementation of the PfSPZ vaccine as an additional intervention alongside existing malaria control measures. This vaccine has the potential to reduce the likelihood of successful transmission from an infected mosquito to a vaccinated individual.

The third key aspect is the possibility of treatment failure among individuals who have undergone malaria treatment. In practice, treatment failure rates can be relatively high in the field. Lastly, only a limited number of studies calibrate their model parameters using real incidence data. Unlike diseases such as dengue, malaria often has a long incubation period, and symptoms in infected individuals may take months to appear, complicating data collection and analysis.

To address these gaps, the current study integrates vaccination using the Plasmodium falciparum sporozoite (PfSPZ) vaccine, indoor residual spraying (IRS), and treatment failure into a unified modeling framework. The model incorporates treatment failure among treated individuals, with its parameters calibrated using malaria incidence data from Keerom, Papua, Indonesia. Keerom is selected as the case study because it represents one of the important malaria-endemic areas in Papua, where malaria incidence remains high compared to the national level. Notably, this study marks the first time that Keerom incidence data have been used for malaria model analysis. Additionally, optimal control and cost-effectiveness analyses are conducted to determine the most effective and cost-effective strategies for controlling and ultimately reducing malaria transmission.

This study is organized as follows. In the next section, we formulate our model as a system of nonlinear ordinary differential equations. The analysis of the model, focusing on the existence and stability of equilibrium points, is presented in Section 3. We demonstrate that the malaria-free equilibrium is always locally stable when the reproduction number is less than one. The existence of the malaria-endemic equilibrium point is implicitly established as a function of the number of infected mosquitoes. We also established that the malaria-endemic equilibrium exists (and is unique) only when the reproduction number exceeds one. Parameter estimation for our model, using incidence data from Keerom, Indonesia, is detailed in Section 4. A sensitivity analysis is performed in Section 5, followed by optimal control simulations in Section 6. Finally, the conclusions are provided in Section 7.

## 2 The formulation of malaria model

### 2.1 Model assumption

As mentioned earlier, this study aims to investigate the impact of the PfSPZ vaccine combined with IRS intervention on malaria control programs. Based on this objective, the model consists of two interacting populations, namely the human and mosquito populations, denoted by *N*(*t*) and *M*(*t*), respectively. We begin the model formulation by defining the compartments of the model as follows:

a Susceptible human compartment, denoted by *S*(*t*). This compartment comprises individuals who are susceptible to malaria and eligible to receive the PfSPZ vaccine intervention.
b Vaccinated human compartment, denoted by *V*(*t*). Susceptible individuals who have already received the PfSPZ vaccine are categorized in this compartment. Even though they have already been vaccinated, they are still able to be infected with malaria due to the imperfect protection of the PfSPZ vaccine.
c The latent compartment, denoted by *L*(*t*), represents newly infected humans who have not yet developed clinical symptoms but are expected to progress to symptomatic infection after the ordinary incubation period. Thus, the latent stage denotes a transitional phase of infection between exposure and clinical malaria (39).
d The dormant compartment, denoted by *D*(*t*), represents infected humans in whom the parasite remains clinically silent or inactive for a longer period before possible activation. Unlike the latent compartment, the dormant compartment is not interpreted as ordinary incubation, but as a delayed activation or relapse-like stage. This assumption is motivated by the dormant hypnozoite stage in *Plasmodium vivax* and *Plasmodium ovale*, which can persist in the liver and later reactivate (40, 41).
e The symptomatic infected humans, denoted by *I*_1_(*t*). This compartment consists of infected individuals who already show clinical symptoms and can transmit malaria if susceptible mosquitoes bite them.
f The symptomatic infected humans who have failed previous treatment, denoted by *I*_2_(*t*). By definition, this compartment consists of infected individuals from *I*_1_(*t*) who have received treatment but failed in the first phase of treatment, causing them to require further malaria treatment.
g The recovered humans, denoted by *R*(*t*). This compartment consists of individuals who have recovered from malaria and have temporary immunity against malaria transmission.
h Susceptible mosquitoes, denoted by *M*_1_(*t*). This compartment consists of mosquitoes that are susceptible to malaria infection. All newborn mosquitoes enter this compartment.
i Infected mosquitoes, denoted by *M*_2_(*t*). This compartment consists of mosquitoes that carry *Plasmodium* parasites and can transmit them to susceptible and vaccinated human compartments through biting.

Based on the above compartment description, the total of human and mosquito populations is then defined as follows:


$$\begin{array}{lll} N\left(t\right) & = & S\left(t\right)+V\left(t\right)+D\left(t\right)+L\left(t\right)+I_{1}\left(t\right)+I_{2}\left(t\right)+R\left(t\right),\;\text{and}\\ M\left(t\right) & = & M_{1}\left(t\right)+M_{2}\left(t\right). \end{array}$$

Before we proceed with our model construction, it is better to introduce our model assumptions.

(i) Malaria is mainly transmitted through the bite of infected female *Anopheles* mosquitoes, while vertical transmission is not considered in this study (42, 43). Hence, we assume that all recruitment from newborns enters the susceptible class. The recruitment rates are denoted by Λ_*h*_ and Λ_*v*_ for the human and mosquito populations, respectively.
(ii) The PfSPZ vaccine is given only to susceptible individuals. We assume that this vaccine reduces the probability of malaria transmission among vaccinated individuals. The vaccination rate for susceptible individuals is denoted by the time-dependent parameter *u*(*t*) (14).
(iii) The PfSPZ vaccine does not confer lifelong protection. Hence, vaccinated individuals may lose their protection and return to the susceptible compartment at a constant rate δ.
(iv) Transmission in the human population is caused by the bite of infected mosquitoes to susceptible and vaccinated individuals, with transmission rates β_1_ and β_2_, respectively. Due to the effect of vaccination, we assume that β_2_ <β_1_. Since PfSPZ has an effectiveness of 78% (14), the value of β_2_ is assumed to be 22% of β_1_.
(v) Infection in susceptible mosquitoes occurs when susceptible mosquitoes bite infected humans in compartments *I*_1_ and *I*_2_, with a constant transmission rate β_3_.
(vi) New infections in humans do not always directly show symptoms. It is possible that newly infected individuals enter a dormant period first before eventually developing symptoms. We use *p* to describe the proportion of newly infected humans who enter the latent period, while 1−*p* represents the proportion of newly infected humans who enter the dormant period.
(vii) Malaria treatment is given only to infected individuals who show symptoms, which in our model are denoted by compartments *I*_1_ and *I*_2_.
(viii) We assume that the recovery rate of infected individuals in *I*_1_ is γ_1_. However, malaria treatment is not always 100% effective, and treated individuals may experience treatment failure (44, 45). Hence, we use *q* to denote the proportion of treated individuals who fail to recover.
(ix) Malaria-induced mortality is not explicitly included in the model because the present study focuses on transmission dynamics and the impact of vaccination and IRS, rather than on mortality burden. Furthermore, the available data consist of reported malaria incidence, whereas reliable malaria-specific mortality data for the study region are unavailable. Hence, malaria-induced death is neglected in our model.
(x) Indoor residual spraying (IRS) is implemented as a vector-control intervention (43, 46). We assume that the IRS does not directly affect the human population but reduces the mosquito population by increasing its mortality rate. Therefore, the natural death rate of mosquitoes is modified from μ_*v*_ to μ_*v*_+*u*_1_(*t*), where *u*_1_(*t*) is the time-dependent IRS intervention.

### 2.2 Model construction

**Figure 1:**
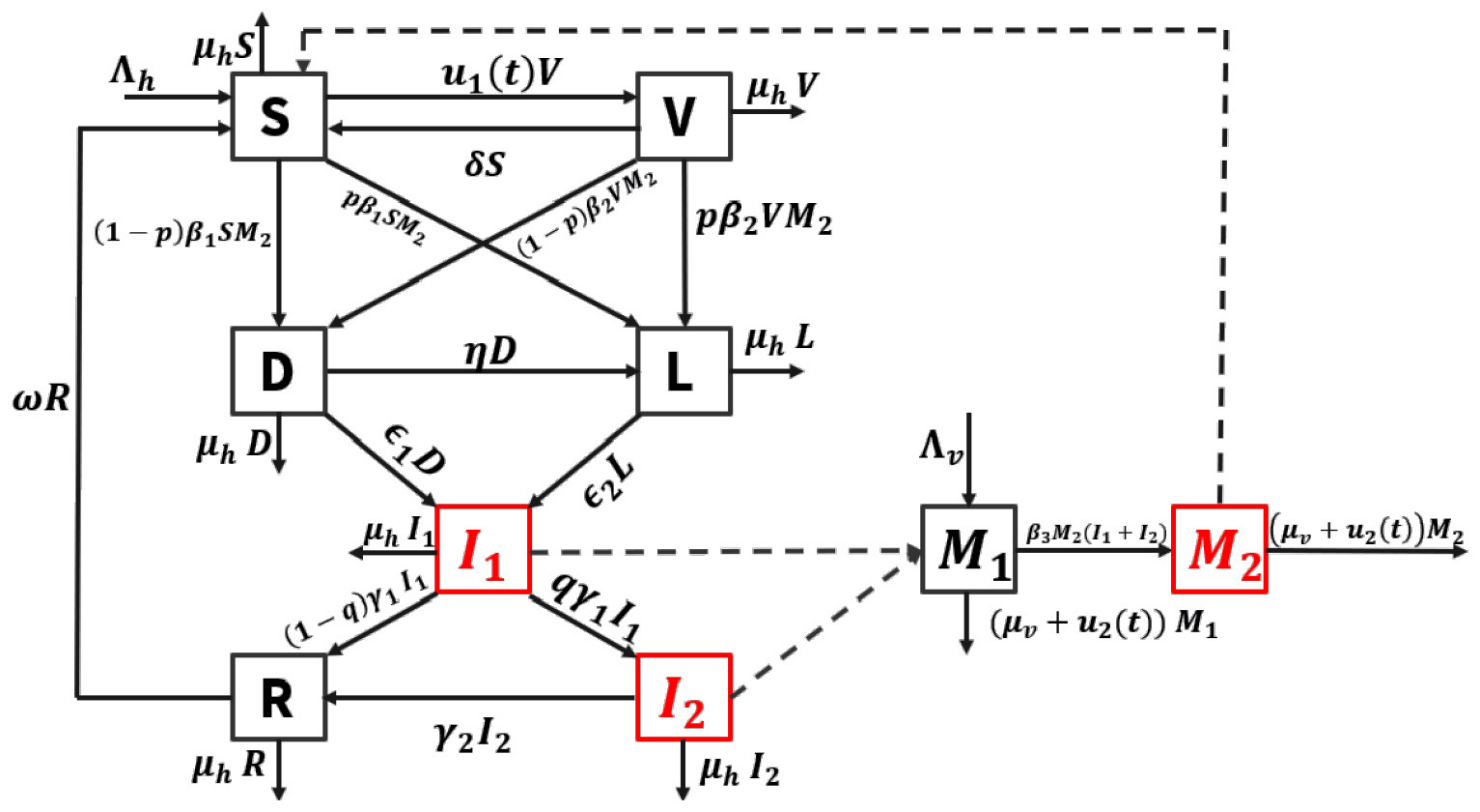
Transmission diagram of malaria model in System Equation 3, with the parameter description are given in Table 1. From the compartmental structure, one of the main novelties of this study is represented by the pathway *S*→*V*→*D*→*L*→*I*_1_→*I*_2_→*R*, which combines PfSPZ vaccination, delayed activation, latent infection, treatment failure, and recovery within a unified malaria transmission framework.

Using previous compartment classification and model assumptions, we are ready to construct our model. Model construction follows the transmission diagram that is given in Figure 1.

#### 2.2.1 Susceptible human dynamics

From our previous assumption, all newborns in the human population always enter the ecosystem through this compartment. Hence, *S*(*t*) increases due to newborn recruitment at a constant rate Λ_*h*_. This compartment decreases due to vaccination intervention with a non-constant rate *u*_1_(*t*), new infection caused by the bite of infected mosquitoes, and the natural death rate μ_*h*_. In this study, malaria transmission between humans and mosquitoes is described using an effective mass-action incidence formulation. Biologically, malaria transmission depends on the mosquito biting rate and the probability of successful transmission from infectious mosquitoes to humans and from infectious humans to mosquitoes. A standard incidence formulation for new human infection can be written as ${\bar{\beta }}_{1}SM_{2}/N_{h}$, where *N*_*h*_ denotes the total human population. Since the total human population is assumed to be constant in this model, the factor 1/*N*_*h*_ can be absorbed into the transmission coefficient. Hence, by defining $\beta_{1}={\bar{\beta }}_{1}/N_{h}$, the incidence term can be written equivalently as β_1_*SM*_2_, where β_1_ is the effective transmission rate incorporating the biting rate, transmission probability, and population-scaling factor. Therefore, the transmission parameter used in this model is interpreted as an effective transmission rate that incorporates the mosquito biting rate, the probability of successful transmission, and a scaling factor due to the constant human population size. Furthermore, this compartment increases due to waning immunity in the *R* and vaccinated compartments, denoted by ω and δ, respectively. Hence, the dynamics of *S*(*t*) satisfy the following equation:


$$\begin{array}{l} \frac{dS}{dt}=\Lambda_{h}-\beta_{1}SM_{2}+\delta V+\omega R-u_{1}\left(t\right)S-\mu_{h}S. \end{array}$$

#### 2.2.2 Vaccinated human dynamics

This compartment increases due to the vaccination of susceptible individuals from the *S*(*t*) compartment at a rate *u*_2_(*t*). It decreases due to new infection β_2_*VM*_2_, waning vaccine-induced protection at a rate δ, and the natural death rate μ_*h*_. Due to imperfect vaccine efficacy, individuals in this compartment may still be infected with malaria at a constant transmission rate β_2_, with β_2_ <β_1_. Hence, the dynamics of *V*(*t*) are modeled as follows:


$$\begin{array}{l} \frac{dV}{dt}=u_{1}\left(t\right)S-\beta_{2}VM_{2}-\delta V-\mu_{h}V. \end{array}$$

#### 2.2.3 Dormant human dynamics

As mentioned before, newly infected individuals from the *S* and *V* compartments do not directly show symptoms. There is a “transition period” during which these newly infected individuals may experience either a dormant or a latency period. Hence, this compartment increases due to new infections from the *S* and *V* compartments, with a proportion of 1−*p*, where 1−*p* denotes the proportion of newly infected individuals entering the *D* compartment. Therefore, the increase of this compartment due to new infection is modeled as (1−*p*)(β_1_*S*+β_2_*V*)*M*_2_. This compartment decreases due to the dormant activation rate η, which moves individuals from the *D* compartment to the *L* compartment, the dormant relapse rate ϵ_1_, and the natural death rate μ_*h*_. Hence, the dynamics of this compartment follow the model below:


$$\begin{array}{l} \frac{dD}{dt}=\left(1-p\right)\left(\beta_{1}S+\beta_{2}V\right)M_{2}-\eta D-\epsilon_{1}D-\mu_{h}D. \end{array}$$

#### 2.2.4 Latent human dynamics

This compartment increases due to newly infected humans from the *S* and *V* compartments who do not experience a dormant period, but directly enter the latent period. This phenomenon is modeled by *p*(β_1_*S*+β_2_*V*)*M*_2_. This compartment also increases due to the dormant activation rate η, which moves individuals from the *D* compartment to the *L* compartment. On the other hand, this compartment decreases due to the natural death rate μ_*h*_ and the latent progression rate ϵ_2_. Therefore, the dynamics of *L*(*t*) are given as follows:


$$\begin{array}{l} \frac{dL}{dt}=p\left(\beta_{1}S\beta_{2}V\right)M_{2}+\eta D-\epsilon_{2}L-\mu_{h}L. \end{array}$$

#### 2.2.5 Symptomatic infected human dynamics

As described before, this compartment consists of infected individuals who have already developed symptoms and can spread malaria to mosquitoes. Therefore, this compartment increases due to progression from the *D* and *L* compartments, denoted by ϵ_1_ and ϵ_2_, respectively. Let the treatment period be denoted by 1/γ_1_. Hence, after this treatment period, individuals from this compartment move to the *R* compartment if they fully recover from malaria, with a proportion 1−*q*, and move to the next stage of infection that requires more comprehensive treatment, represented by the *I*_2_ compartment, with a proportion *q*. Due to the natural death rate μ_*h*_, this compartment satisfies the following equation:


$$\begin{array}{l} \frac{dI_{1}}{dt}=\epsilon_{1}D+\epsilon_{2}L-\left(1-q\right)\gamma_{1}I_{1}-q\gamma_{1}I_{2}-\mu_{h}I_{1}. \end{array}$$

#### 2.2.6 Symptomatic infected human dynamics who follow second base treatment

This compartment consists of individuals who can transmit the disease, and failed in the first stage of treatment. Therefore, this compartment increases due to infected individuals in *I*_1_ who fail treatment at rate *qγ*_1_, and decreases due to the recovery rate γ_2_ and the natural death rate μ_*h*_. Hence, the dynamic of *I*_2_ is as follows:


$$\begin{array}{l} \frac{dI_{2}}{dt}=q\gamma_{1}I_{1}-\gamma_{2}I_{2}-\mu_{h}I_{2}. \end{array}$$

#### 2.2.7 Recovered human dynamics

This compartment increases due to newly recovered individuals from the *I*_1_ and *I*_2_ compartments, and decreases due to loss of temporary immunity at a constant rate ω and the natural death rate μ_*h*_. Hence, we have


$$\begin{array}{l} \frac{dR}{dt}=\left(1-q\right)\gamma_{1}I_{1}+\gamma_{2}I_{2}-\omega R-\mu_{h}R. \end{array}$$

#### 2.2.8 Susceptible mosquitoes dynamics

Based on our first assumption in the previous section, all newborn mosquitoes enter the ecosystem through the susceptible mosquito compartment at a constant rate Λ_*v*_. This compartment decreases due to new infections from bites by infected individuals in the *I*_1_ and *I*_2_ compartments, with a constant transmission rate β_3_. It also decreases due to the natural death rate μ_*v*_ and IRS intervention at a rate *u*_2_(*t*). Hence, the dynamics of susceptible mosquitoes are given by


$$\begin{array}{l} \frac{dM_{1}}{dt}=\Lambda_{v}-\beta_{3}M_{1}\left(I_{1}+I_{2}\right)-\left(\mu_{v}+u_{2}\left(t\right)\right)M_{1}. \end{array}$$

#### 2.2.9 Infected mosquitoes dynamics

This compartment increases due to new infection from the susceptible mosquitoes compartment. It decreases for the same reason as *M*_1_, i.e., due to the natural death rate μ_*v*_ and the IRS intervention *u*_2_(*t*). Hence, we have the dynamic of *M*_1_ following:


$$\begin{array}{l} \frac{dM_{2}}{dt}=\beta_{3}M_{1}\left(I_{1}+I_{2}\right)-\left(\mu_{v}+u_{2}\left(t\right)\right)M_{2}. \end{array}$$

Based on the mentioned explanation on our model construction, the mathematical model for malaria transmission involving vaccination, treatment failure, relapse, and IRS is given by the following set of differential equations:


$$\begin{array}{l} \frac{dS}{dt}=\Lambda_{h}-\left(1-p\right)\beta_{1}SM_{2}-p\beta_{1}SM_{2}+\delta V+\omega R\\ \;-u_{1}\left(t\right)S-\mu_{h}S,\\ \frac{dV}{dt}=u_{1}\left(t\right)S-\left(1-p\right)\beta_{2}VM_{2}-p\beta_{2}VM_{2}-\delta V-\mu_{h}V,\\ \frac{dD}{dt}=\left(1-p\right)\beta_{1}SM_{2}+\left(1-p\right)\beta_{2}VM_{2}-\eta D-\epsilon_{1}D-\mu_{h}D,\frac{dL}{dt}=p\beta_{1}SM_{2}+p\beta_{2}VM_{2}+\eta D-\epsilon_{2}L-\mu_{h}L,\frac{dI_{1}}{dt}=\epsilon_{1}D+\epsilon_{2}L-\left(1-q\right)\gamma_{1}I_{1}-q\gamma_{1}I_{1}-\mu_{h}I_{1},\\ \frac{dI_{2}}{dt}=q\gamma_{1}I_{1}-\gamma_{2}I_{2}-\mu_{h}I_{2},\\ \frac{dR}{dt}=\left(1-q\right)\gamma_{1}I_{1}+\gamma_{2}I_{2}-\omega R-\mu_{h}R,\\ \frac{dM_{1}}{dt}=\Lambda_{v}-\beta_{3}M_{1}\left(I_{1}+I_{2}\right)-\left(\mu_{v}+u_{2}\left(t\right)\right)M_{1},\\ \frac{dM_{2}}{dt}=\beta_{3}M_{1}\left(I_{1}+I_{2}\right)-\left(\mu_{v}+u_{2}\left(t\right)\right)M_{2}, \end{array}$$ (1)

subject to the initial conditions *S*>0, *V*≥0, *D*≥0, *L*≥0, *I*_1_≥0, *I*_2_≥0, *R*≥0, *M*_1_>0, *M*_2_≥0. Our aim is to reduce the number of the infected individuals, *I*_1_ and *I*_2_ by implementing an optimal rate of *u*_1_(*t*) and *u*_2_(*t*). Mathematically, our objective can be formulated by minimizing the following cost function:


$$\begin{array}{l} J\left(u_{1},u_{2},X\right)=\int_{0}^{T}\left(\omega_{1}I_{1}\left(t\right)+\omega_{2}I_{2}\left(t\right)+c_{1}{\left(u_{1}\left(t\right)\right)}^{2}+c_{2}{\left(u_{2}\left(t\right)\right)}^{2}\right)dt, \end{array}$$ (2)

where ω_1_ and ω_2_ are the weights of the objective function for *I*_1_ and *I*_2_, respectively, *c*_1_ and *c*_2_ are the weight coefficient for control variable *u*_1_(*t*) and *u*_2_(*t*), respectively, *X* as the set of infected compartment that we want to minimize, while *T* is the final time of simulation.

In Sections 3–5, we analyze the non-autonomous version of our proposed model in Equation 3 by assuming *u*_1_(*t*) = *u*_1_ and *u*_2_(*t*) = *u*_2_. Optimal control analysis will be discussed in Section 6.

## 3 Mathematical model analysis

A mathematical analysis of the non-autonomous version of the malaria model in Equation 3 is discussed in this section. A preliminary analysis is conducted to establish the biologically well-defined properties of the model, including the existence and positivity of solutions. Furthermore, the existence and local stability criteria of the equilibrium points are analyzed and connected to the basic reproduction number of the proposed model.

By assuming the control rate *u*_1_(*t*) and *u*_2_(*t*) are constant in time, we have the non-autonomous version of system Equation 3 is given by:


$$\begin{array}{l} \frac{dS}{dt}=\Lambda_{h}-\left(1-p\right)\beta_{1}SM_{2}-p\beta_{1}SM_{2}+\delta V+\omega R-u_{1}S-\mu_{h}S,\\ \frac{dV}{dt}=u_{1}S-\left(1-p\right)\beta_{2}VM_{2}-p\beta_{2}VM_{2}-\delta V-\mu_{h}V,\\ \frac{dD}{dt}=\left(1-p\right)\beta_{1}SM_{2}+\left(1-p\right)\beta_{2}VM_{2}-\eta D-\epsilon_{1}D-\mu_{h}D,\frac{dL}{dt}=p\beta_{1}SM_{2}+p\beta_{2}VM_{2}+\eta D-\epsilon_{2}L-\mu_{h}L,\frac{dI_{1}}{dt}=\epsilon_{1}D+\epsilon_{2}L-\left(1-q\right)\gamma_{1}I_{1}-q\gamma_{1}I_{1}-\mu_{h}I_{1},\\ \frac{dI_{2}}{dt}=q\gamma_{1}I_{1}-\gamma_{2}I_{2}-\mu_{h}I_{2},\\ \frac{dR}{dt}=\left(1-q\right)\gamma_{1}I_{1}+\gamma_{2}I_{2}-\omega R-\mu_{h}R,\\ \frac{dM_{1}}{dt}=\Lambda_{v}-\beta_{3}M_{1}\left(I_{1}+I_{2}\right)-\left(\mu_{v}+u_{2}\right)M_{1},\\ \frac{dM_{2}}{dt}=\beta_{3}M_{1}\left(I_{1}+I_{2}\right)-\left(\mu_{v}+u_{2}\right)M_{2}. \end{array}$$ (3)

### 3.1 Preliminary analysis

**Lemma 1**. Suppose that *S*(*t*), *V*(*t*), *D*(*t*), *L*(*t*), *I*_1_(*t*), *I*_2_(*t*), *R*(*t*), *M*_1_(*t*), and *M*_2_(*t*) are continuous and non-negative for all *t*≥0. Furthermore, let all the initial conditions for each variable in system Equation 3 be non-negative. Then the malaria model in system Equation 3 admits a global solution *S*(*t*), *V*(*t*), *D*(*t*), *L*(*t*), *I*_1_(*t*), *I*_2_(*t*), *R*(*t*), *M*_1_(*t*), and *M*_2_(*t*) for all *t*≥0, and it will always remain non-negative for all *t*≥0. Moreover,


$$\begin{array}{l} N\left(t\right)\leq \max\left\{N\left(0\right),\frac{\Lambda_{h}}{\mu_{h}}\right\},\;M\left(t\right)\leq \max\left\{M\left(0\right),\frac{\Lambda_{v}}{\mu +u_{2}}\right\}. \end{array}$$ (4)

Proof. Let $Y={\left(S,V,D,L,I_{1},I_{2},R,M_{1},M_{2}\right)}^{T}$. The right-hand side of system Equation 3 is polynomial in the state variables, hence continuous and locally Lipschitz on ℝ^9^. Therefore, by the Picard–Lindelf theorem, for every non-negative initial condition, there exists a unique local solution.

For the positivity of the solutions, define


$$\begin{array}{l} \Omega ={\mathbb{R}}_{+}^{9}\\ \;=\left\{\left(S,V,D,L,I_{1},I_{2},R,M_{1},M_{2}\right)\in {\mathbb{R}}^{9}:\text{all components are}\geq 0\right\}. \end{array}$$

We show Ω is positively invariant by checking the sign of each derivative on the corresponding boundary. From direct calculation, we have:


$$\begin{array}{l} \;\frac{dS}{dt}{|}_{S=0}=\Lambda_{h}+\delta V+\omega R,\\ \;\frac{dV}{dt}{|}_{V=0}=u_{1}S,\\ \;\frac{dD}{dt}{|}_{D=0}=\left(1-p\right)M_{2}\left(\beta_{1}S+\beta_{2}V\right),\\ \;\frac{dL}{dt}{|}_{L=0}=pM_{2}\left(\beta_{1}S+\beta_{2}V\right),\\ \;\frac{dI_{1}}{dt}{|}_{I_{1}=0}=\epsilon_{1}D+\epsilon_{2}L,\\ \;\frac{dI_{2}}{dt}{|}_{I_{2}=0}=q\gamma_{1}I_{1},\\ \;\frac{dR}{dt}{|}_{R=0}=\left(1-q\right)\gamma_{1}I_{1}+\gamma_{2}I_{2},\\ \frac{dM_{1}}{dt}{|}_{M_{1}=0}=\Lambda_{v},\\ \frac{dM_{2}}{dt}{|}_{M_{2}=0}=\beta_{3}M_{1}\left(I_{1}+I_{2}\right). \end{array}$$

Hence, on every boundary hyperplane of Ω, the vector field points inward or is tangent. Therefore, Ω is positively invariant. So if all initial data are non-negative, then all variables will be non-negative for all *t*≥0.

For the boundedness of the human and mosquito population, adding the first seven equations in system Equation 3, we have:


$$\begin{array}{l} \frac{dN}{dt}=\Lambda_{h}-\mu_{h}N, \end{array}$$

which implies $N\left(t\right)\leq \max\left\{N\left(0\right),\frac{\Lambda_{h}}{\mu_{h}}\right\}$. With a similar approach, adding the last two equations in system Equation 3, we have:


$$\begin{array}{l} \frac{dM}{dt}=\Lambda_{v}-\left(\mu_{v}+u_{2}\right)M, \end{array}$$

which implies $M\left(t\right)\leq \max\left\{M\left(0\right),\frac{\Lambda_{v}}{\mu +u_{2}}\right\}.$ Thus all solutions are bounded, and therefore the local solution extends globally. This completes the proof.

### 3.2 Malaria-free equilibrium and the basic reproduction number

The malaria-free equilibrium point (MFE) is the condition in which malaria is no longer present in a population. Based on this definition, the malaria-free equilibrium point for system Equation 3 is given by,


$$\begin{array}{l} \;DFE\\ \;=\left(S^{0},V^{0},D^{0},L^{0},I_{1}^{0},I_{2}^{0},R^{0},M_{1}^{0},M_{2}^{0}\right)\\ =\left(\frac{\left(\delta +\mu_{h}\right)\Lambda_{h}}{\left(\delta +u_{1}+\mu_{h}\right)\mu_{h}},\frac{\Lambda_{h}u_{1}}{\left(\delta +u_{1}+\mu_{h}\right)\mu_{h}},0,0,0,0,0,\frac{\Lambda_{v}}{u_{2}+\mu_{v}},0\right). \end{array}$$

Next, we calculate the basic reproduction number of our proposed model. Many authors have used the basic reproduction number as the endemic threshold of their proposed vector-borne model, such as in Aldila et al. (21, 47–49). They found that the disease will go extinct if the basic reproduction number is less than 1, and will persist if it is greater than 1. The basic reproduction number is determined from the spectral radius of the next-generation matrix of the respective model. We use the next-generation matrix approach, following the formula introduced in Diekmann et al. (50), to determine the basic reproduction number of the system Equation 3. The transition matrix (*V*) and transmission matrix (*F*) of System Equation 3 are given by


$$\begin{array}{ll} V & =\left[\begin{array}{ccccc} -\eta -\mu_{h}-\epsilon_{1} & 0 & 0 & 0 & 0\\ \eta & -\epsilon_{2}-\mu_{h} & 0 & 0 & 0\\ \epsilon_{1} & \epsilon_{2} & -q\gamma_{1}-\left(1-q\right)\gamma_{1}-\mu_{h} & 0 & 0\\ 0 & 0 & q\gamma_{1} & -\gamma_{2}-\mu_{h} & 0\\ 0 & 0 & 0 & 0 & -u_{2}-\mu_{v} \end{array}\right],\\ F & =\left[\begin{array}{ccccc} 0 & 0 & 0 & 0 & \frac{\left(1-p\right)\beta_{1}\left(\delta +\mu_{h}\right)\Lambda_{h}}{\mu_{h}\left(\delta +\mu_{h}+u_{1}\right)}+\frac{\left(1-p\right)\beta_{2}\Lambda_{h}u_{1}}{\mu_{h}\left(\delta +\mu_{h}+u_{1}\right)}\\ 0 & 0 & 0 & 0 & \frac{p\beta_{1}\left(\delta +\mu_{h}\right)\Lambda_{h}}{\mu_{h}\left(\delta +\mu_{h}+u_{1}\right)}+\frac{p\beta_{2}\Lambda_{h}u_{1}}{\mu_{h}\left(\delta +\mu_{h}+u_{1}\right)}\\ 0 & 0 & 0 & 0 & 0\\ 0 & 0 & 0 & 0 & 0\\ 0 & 0 & \frac{\beta_{3}\Lambda_{v}}{u_{2}+\mu_{v}} & \frac{\beta_{3}\Lambda_{v}}{u_{2}+\mu_{v}} & 0 \end{array}\right]. \end{array}$$

Using the formula *NGM* = −*E*′*FV*^−1^*E*, where


$$\begin{array}{l} E=\left[\begin{array}{ccc} 1 & 0 & 0\\ 0 & 1 & 0\\ 0 & 0 & 0\\ 0 & 0 & 0\\ 0 & 0 & 1 \end{array}\right], \end{array}$$

and *E*′ is the transpose of *E*, we have the basic reproduction number, which is taken from the spectral radius of *NGM* is given by,


$$R_{0}=\sqrt{\frac{\beta_{3}\Lambda_{h}\Lambda_{v}(\delta \beta_{1}+\mu_{h}\beta_{1}+\beta_{2}u_{1})(\left(1-p)\epsilon_{1}\mu_{h}+\epsilon_{2}(p\mu_{h}+\eta +\epsilon_{1}\right))(q\gamma_{1}+\gamma_{2}+\mu_{h})}{\mu_{h}(\delta +\mu_{h}+u_{1}){\left(u_{2}+\mu_{v}\right)}^{2}(\eta +\mu_{h}+\epsilon_{1})(\gamma_{1}+\mu_{h})(\gamma_{2}+\mu_{h})(\epsilon_{2}+\mu_{h})}}.$$ (5)

Since our model satisfies the five assumptions outlined in van den Driessche and Watmough (51) (see Appendix A), we can state the following theorem regarding the stability of the malaria-free equilibrium point.

**Theorem 1**. The malaria-free equilibrium of system (MFE) (Equation 3) is always locally asymptotically stable if $R_{0}<1$ and unstable if $R_{0}>1$.

### 3.3 Existence of malaria-endemic equilibrium

The malaria endemic equilibrium (MEE) point of system (Equation 3) is given by,


$$\begin{array}{l} EE=\left(S^{*},V^{*},D^{*},L^{*},I_{1}^{*},I_{2}^{*},R^{*},M_{1}^{*},M_{2}^{*}\right), \end{array}$$

where


$$\begin{array}{cc} S^{*} & =\frac{I_{1}^{*}\left(\mu_{h}+\gamma_{1}\right)\left(\mu_{h}+\epsilon_{2}\right)\left(\eta +\mu_{h}+\epsilon_{1}\right)\left(M_{2}^{*}\beta_{2}+\delta +\mu_{h}\right)}{M_{2}^{*}\left[\epsilon_{2}\left(\eta +\epsilon_{1}+\mu_{h}\right)+\epsilon_{1}\mu_{h}\left(1-p\right)+p\epsilon_{2}\mu_{h}\right]\left[M_{2}^{*}\beta_{1}\beta_{2}+\beta_{1}\left(\delta +\mu_{h}\right)+\beta_{2}u_{1}\right]},\\ V^{*} & =\frac{I_{1}^{*}u_{1}\left(\mu_{h}+\gamma_{1}\right)\left(\mu_{h}+\epsilon_{2}\right)\left(\eta +\mu_{h}+\epsilon_{1}\right)}{M_{2}^{*}\left[\epsilon_{2}\left(\eta +\epsilon_{1}+\mu_{h}\right)+\epsilon_{1}\mu_{h}\left(1-p\right)+p\epsilon_{2}\mu_{h}\right]\left[M_{2}^{*}\beta_{1}\beta_{2}+\beta_{1}\left(\delta +\mu_{h}\right)+\beta_{2}u_{1}\right]},\\ D^{*} & =\frac{I_{1}^{*}\left(\mu_{h}+\gamma_{1}\right)\left(\mu_{h}+\epsilon_{2}\right)\left(1-p\right)}{p\epsilon_{2}\mu_{h}+\eta \epsilon_{2}+\epsilon_{1}\epsilon_{2}+\left(1-p\right)\epsilon_{1}\mu_{h}},\\ L^{*} & =\frac{I_{1}^{*}\left(\mu_{h}+\gamma_{1}\right)\left(p\epsilon_{1}+p\mu_{h}+\eta \right)}{p\epsilon_{2}\mu_{h}+\eta \epsilon_{2}+\epsilon_{1}\epsilon_{2}+\left(1-p\right)\epsilon_{1}\mu_{h}},\\ I_{1}^{*} & =\frac{\epsilon_{1}D^{*}+\epsilon_{2}L^{*}}{\gamma_{1}+\mu_{h}},\\ I_{2}^{*} & =\frac{q\gamma_{1}I_{1}^{*}}{\gamma_{2}+\mu_{h}},\\ R^{*} & =\frac{I_{1}^{*}\gamma_{1}\left(\gamma_{2}+\left(1-q\right)\mu_{h}\right)}{\left(\mu_{h}+\gamma_{2}\right)\left(\mu_{h}+\omega \right)},\\ M_{1}^{*} & =\frac{M_{2}^{*}\left(\mu_{v}+u_{2}\right)\left(\mu_{h}+\gamma_{2}\right)}{I_{1}^{*}\beta_{3}\left(q\gamma_{1}+\gamma_{2}+\mu_{h}\right)}, \end{array}$$

with $M_{2}^{*}$ is taken from the positive roots of the following polynomial:


$$F(M_{2})=p_{2}M_{2}^{2}+p_{1}M_{2}+p_{0}=0,$$ (6)

with *p*_2_ = β_1_β_2_(μ_*v*_+*u*_2_)Φ, where


$$\begin{array}{l} \Phi =pq\gamma_{1}\mu_{h}\left(\epsilon_{2}-\epsilon_{1}\right)K+p\mu_{h}\left(\epsilon_{2}-\epsilon_{1}\right)\left(\gamma_{2}+\mu_{h}\right)L\\ \;+q\gamma_{1}\left(\epsilon_{1}\epsilon_{2}+\epsilon_{1}\mu_{h}+\eta \epsilon_{2}\right)K+\left(\gamma_{2}+\mu_{h}\right)\\ \;\left[M+\mu_{h}\left(\mu_{v}+u_{2}\right)N\right], \end{array}$$ (7)

while


$$\begin{array}{lll} K & = & \Lambda_{h}\beta_{3}\left(\omega +\mu_{h}\right)+\omega \mu_{h}\left(\mu_{v}+u_{2}\right), \end{array}$$ (8)

$$\begin{array}{lll} L & = & \Lambda_{h}\beta_{3}\left(\omega +\mu_{h}\right)-\omega \gamma_{1}\left(\mu_{v}+u_{2}\right), \end{array}$$ (9)

$$\begin{array}{lll} M & = & \Lambda_{h}\beta_{3}\left(\omega +\mu_{h}\right)\left(\epsilon_{1}\epsilon_{2}+\epsilon_{1}\mu_{h}+\eta \epsilon_{2}\right), \end{array}$$ (10)

$$\begin{array}{l} N=\left(\gamma_{1}+\mu_{h}+\omega \right)\left(\epsilon_{1}\epsilon_{2}+\epsilon_{1}\mu_{h}+\eta \epsilon_{2}\right)\\ \;+\left(\epsilon_{2}+\eta +\mu_{h}\right)\left(\gamma_{1}+\mu_{h}\right)\left(\omega +\mu_{h}\right), \end{array}$$ (11)

and $p_{0}=\left(1-R_{0}^{2}\right){\left(\mu_{v}+u_{2}\right)}^{2}\left(\gamma_{2}+\mu_{h}\right)\left(\gamma_{1}+\mu_{h}\right)\left(\epsilon_{2}+\mu_{h}\right)$ (η+ϵ_1_+μ_*h*_)(δ+μ_*h*_+*u*_1_)μ_*h*_(ω+μ_*h*_). Although the expression of *p*_1_ is too complicated to be presented in this article, our numerical experiments suggest that *p*_1_ is always positive for the parameter values considered. From this, we observe that if $R_{0}^{2}>1$, then *p*_0_ <0. Hence, polynomial (Equation 6) has a unique positive root when $R_{0}>1$, which indicates the existence of a unique endemic equilibrium for $R_{0}>1$. This result is stated in the following theorem.

**Theorem 2**. Assume that *p*_1_>0. Then, system (Equation 3) has a unique endemic equilibrium *MEE* if $R_{0}>1$, and has no endemic equilibrium otherwise.

**Figure 2:**
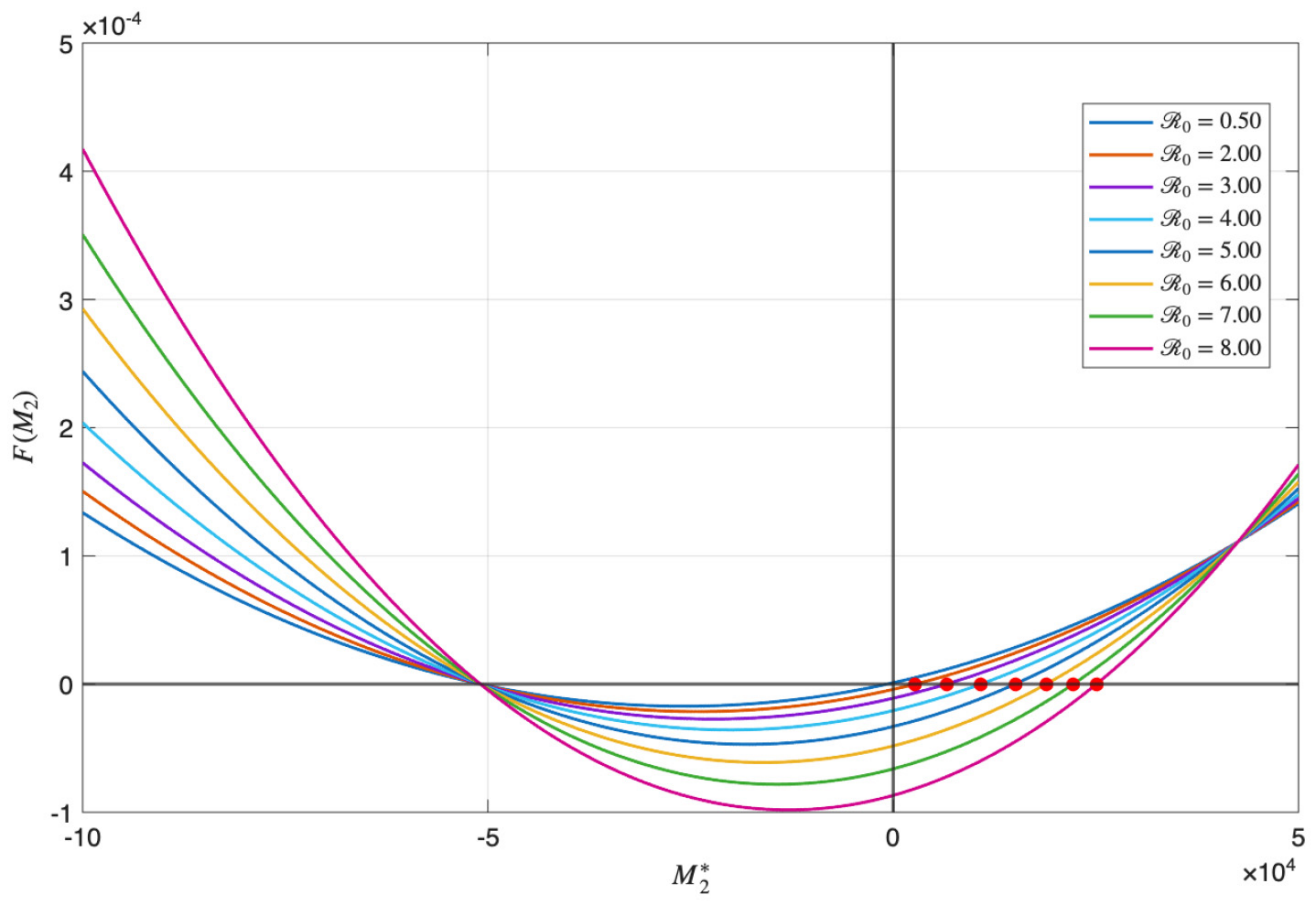
Existence of unique positive roots of *F*(*M*_2_) = 0 for various values of $R_{0}>1$.

To illustrate the existence of a unique positive root of *F*(*M*_2_) for various values of $R_{0}$, we perform a numerical experiment, as shown in Figure 2, using the parameter values in Table 1. Substituting these parameter values into Equation 6, we obtain


$$\begin{array}{l} F\left(M_{2}\right)=2.08\times 10^{-10}\left(0.00013+3.04\times 10^{-6}R_{0}^{2}\right)M_{2}^{2}\\ \;+\left(5.33\times 10^{-12}R_{0}^{2}+1.41\times 10^{-9}\right)M_{2}\\ \;+1.37\times 10^{-6}\left(1-R_{0}^{2}\right). \end{array}$$

**Table 1:** The parameters of malaria model in Equation 3.

Par	Description	Units	Value	Ref
Λ_*h*_	Natural recruitment rate of human population	$\frac{\text{human}}{\text{month}}$	$\frac{62,777}{71.5\times 12}$	(64, 65)
Λ_*v*_	Natural recruitment rate of mosquito	$\frac{\text{mosquito}}{\text{month}}$	$\frac{2\times 62,777\times 30}{21}$	(64, 66)
μ_*h*_	Natural human death rate	$\frac{1}{\text{month}}$	$\frac{1}{71.5\times 12}$	(65)
μ_*v*_	Natural mosquito death rate	$\frac{1}{\text{month}}$	$\frac{30}{21}$	(66)
γ_1_	Recovery rate of *I*_1_	$\frac{1}{\text{month}}$	0.8766	Fitted
γ_2_	Recovery rate of *I*_2_	$\frac{1}{\text{month}}$	0.00075	Fitted
β_1_	Average probability of transmission success rate from mosquito to human in S	$\frac{\text{human}}{\text{mosq}\times \text{month}}$	1.7849 × 10^−5^	Fitted
β_2_	Average probability of transmission success rate from mosquito to human in V	$\frac{\text{human}}{\text{mosq}\times \text{month}}$	3.3 × 10^−6^	Estimated
β_3_	biting rate from mosquito to human	$\frac{1}{\text{month}}$	7.5 × 10^−6^	Fitted
*p*	Proportion of exposed individuals who do not experience dormant period	-	0.99	Fitted
*q*	Proportion of treated individuals who experience recrudescence (treatment failure)	-	0.08625	Fitted
δ	waning efficacy rate of the vaccine	$\frac{1}{\text{month}}$	$\frac{1}{6}$	(19)
*u*_1_	Rate of vaccination	$\frac{1}{\text{month}}$	0.001	(19)
*u*_2_	Rate of IRS	$\frac{1}{\text{month}}$	3.425	Estimated
η	relapse rate	$\frac{1}{\text{month}}$	0.44375	Fitted
ϵ_1_	progress rate from D to *I*_1_	$\frac{1}{\text{month}}$	0.215	Fitted
ϵ_2_	progress rate from L to *I*_1_	$\frac{1}{\text{month}}$	5.865	Fitted
ω	Rate of loss of natural immunity for human population	$\frac{1}{\text{month}}$	0.062	Fitted

Some parameter values are taken from the parameter estimation results in Section 4. Parameters labeled as “fitted” were obtained through the curve-fitting procedure described in Section 4. Conversely, parameters labeled as “estimated” were assigned based on biological assumptions and epidemiological interpretation. These parameters could not be fitted directly because the available field data do not yet include the implementation of a vaccination strategy. Moreover, these values could not be taken directly from previous literature, since, to the best of our knowledge, no existing mathematical modeling study has specifically incorporated the PfSPZ vaccine in this context.

We note that *F*(*M*_2_) is a quadratic function of *M*_2_, with leading coefficient $p_{2}\left(R_{0}\right)>0$. Hence, each curve of *F*(*M*_2_) with respect to *M*_2_ is an upward-opening parabola. For $R_{0}>1$, we have *p*_0_ <0, which implies that *F*(0) <0, while *F*(*M*_2_) → ∞ as *M*_2_ → ∞. Therefore, each curve must intersect the horizontal axis exactly once for *M*_2_>0. This confirms the existence of a unique positive root, which corresponds to the unique endemic equilibrium. This behavior is clearly illustrated in Figure 2, where for each $R_{0}>1$, the quadratic curve intersects the *M*_2_-axis at exactly one positive point.

A more rigorous result regarding the existence of the endemic equilibrium for $R_{0}>1$ is presented in the following theorem. Furthermore, whenever it exists, it is always stable.

**Theorem 3**. The malaria model in system Equation 3 always exhibits a transcritical bifurcation at $R_{0}=1$.

Proof. To prove the theorem, we use the well-known bifurcation theorem by the authors in Song et al. (52). To use the results by the author, first, we redefine our model symbols as follows:


$$\begin{array}{lll} x_{1} & = & S,\;x_{2}=V,\;x_{3}=D,\;x_{4}=L,\;x_{5}=I_{1},\;x_{6}=I_{2},\\ x_{7} & = & R,\;x_{8}=M_{1},\;x_{9}=M_{2},\\ g_{1} & = & \frac{dS}{dt},\;g_{2}=\frac{dV}{dt},\;g_{3}=\frac{dD}{dt},\;g_{4}=\frac{dL}{dt},\;g_{5}=\frac{dI_{1}}{dt},\\ g_{6} & = & \frac{dI_{2}}{dt},\;g_{7}=\frac{dR}{dt},\\ g_{8} & = & \frac{dM_{1}}{dt},\;\text{and }g_{9}=\frac{dM_{2}}{dt}. \end{array}$$

Next, we choose β_1_ as the bifurcation parameter. Hence, we express β_1_ as a function of $R_{0}$ and evaluate it at $R_{0}=1$. From direct calculation, we have


$$\begin{array}{cc} \beta_{1}^{*} & =\frac{{\left(u_{2}+\mu_{v}\right)}^{2}\mu_{h}^{6}{\left(u_{2}+\mu_{v}\right)}^{2}\left(\epsilon_{1}+\epsilon_{2}+\eta +\delta +\gamma_{1}+\gamma_{2}+u_{1}\right)\mu_{h}^{5}+b_{4}\mu_{h}^{4}+b_{3}\mu_{h}^{3}+b_{2}\mu_{h}^{2}+b_{1}\mu_{h}+b_{0}}{\left(\left(p\epsilon_{2}+\epsilon_{1}\left(1-p\right)\right)\mu_{h}+\epsilon_{2}\left(\epsilon_{1}+\eta \right)\right)\Lambda_{v}\beta_{3}\Lambda_{h}\left(q\gamma_{1}+\gamma_{2}+\mu_{h}\right)\left(\delta +\mu_{h}\right)}. \end{array}$$

with


$$\begin{array}{l} b_{4}=(\left(\epsilon_{1}+\eta +\delta +\gamma_{1}+\gamma_{2}+u_{1}\right)\epsilon_{2}+\left(\epsilon_{1}+\eta +\delta +\gamma_{2}+u_{1}\right)\gamma_{1}\\ \;+\left(\epsilon_{1}+\eta +\delta +u_{1}\right)\gamma_{2}+\left(\delta +u_{1}\right)\left(\epsilon_{1}+\eta \right)){\left(u_{2}+\mu_{v}\right)}^{2},\\ b_{3}={\left(u_{2}+\mu_{v}\right)}^{2}(\left(\left(\epsilon_{1}+\eta +\delta +\gamma_{2}+u_{1}\right)\gamma_{1}+\left(\epsilon_{1}+\eta +\delta +u_{1}\right)\gamma_{2}+\left(\delta +u_{1}\right)\left(\epsilon_{1}+\eta \right)\right)\epsilon_{2}\\ \;+\left(\left(\epsilon_{1}+\eta +\delta +u_{1}\right)\gamma_{2}+\left(\delta +u_{1}\right)\left(\epsilon_{1}+\eta \right)\right)\gamma_{1}+\gamma_{2}\left(\delta +u_{1}\right)\left(\epsilon_{1}+\eta \right)),\\ b_{2}=(\left(\left(\epsilon_{1}+\eta +\delta +u_{1}\right)\gamma_{2}+\left(\delta +u_{1}\right)\left(\epsilon_{1}+\eta \right)\right)\gamma_{1}+\gamma_{2}\left(\delta +u_{1}\right)\left(\epsilon_{1}+\eta \right))\epsilon_{2}\mu_{v}^{2}\\ \;+2u_{2}(\left(\left(\epsilon_{1}+\eta +\delta +u_{1}\right)\gamma_{2}+\left(\delta +u_{1}\right)\left(\epsilon_{1}+\eta \right)\right)\gamma_{1}+\gamma_{2}\left(\delta +u_{1}\right)\left(\epsilon_{1}+\eta \right))\epsilon_{2}\mu_{v}\\ \;+(\left(\left(\epsilon_{1}+\eta +\delta +u_{1}\right)\gamma_{2}+\left(\delta +u_{1}\right)\left(\epsilon_{1}+\eta \right)\right)\gamma_{1}+\gamma_{2}\left(\delta +u_{1}\right)\left(\epsilon_{1}+\eta \right))\epsilon_{2}u_{2}^{2}\\ \;+\beta_{2}\left(p\epsilon_{2}+\epsilon_{1}\left(1-p\right)\right)\Lambda_{v}u_{1}\beta_{3}\Lambda_{h},\\ b_{1}=\epsilon_{2}\gamma_{1}\gamma_{2}\left(\delta +u_{1}\right)\left(\epsilon_{1}+\eta \right)\mu_{v}^{2}+2\epsilon_{2}\gamma_{1}\gamma_{2}u_{2}\left(\delta +u_{1}\right)\left(\epsilon_{1}+\eta \right)\mu_{v}\\ \;+\epsilon_{2}\gamma_{1}\gamma_{2}\left(\delta +u_{1}\right)\left(\epsilon_{1}+\eta \right)u_{2}^{2}\\ \;+\beta_{2}\Lambda_{v}u_{1}\beta_{3}(\left(pq\gamma_{1}+p\gamma_{2}+\eta +\epsilon_{1}\right)\epsilon_{2}+\epsilon_{1}\left(q\gamma_{1}+\gamma_{2}\right)\left(1-p\right)\Lambda_{h}),\\ b_{0}=\beta_{2}\beta_{3}\Lambda_{h}\Lambda_{v}\epsilon_{2}u_{1}\left(q\gamma_{1}+\gamma_{2}\right)\left(\epsilon_{1}+\eta \right). \end{array}$$

Next, we analyze the existence of a simple zero eigenvalue of the Jacobian matrix of system Equation 3 evaluated at $\beta_{1}=\beta_{1}^{*}$ and *MFE*. By reordering the variables as


$$\begin{array}{c} \left(D,L,I_{1},I_{2},S,V,R,M_{1},M_{2}\right), \end{array}$$

instead of


$$\begin{array}{c} \left(S,V,D,L,I_{1},I_{2},R,M_{1},M_{2}\right). \end{array}$$

we reveal the Jacobian matrix A as a block structure. That is, we use the permutation


$$\begin{array}{c} \Pi =\left[\begin{array}{ccccccccc} 0 & 0 & 1 & 0 & 0 & 0 & 0 & 0 & 0\\ 0 & 0 & 0 & 1 & 0 & 0 & 0 & 0 & 0\\ 0 & 0 & 0 & 0 & 1 & 0 & 0 & 0 & 0\\ 0 & 0 & 0 & 0 & 0 & 1 & 0 & 0 & 0\\ 1 & 0 & 0 & 0 & 0 & 0 & 0 & 0 & 0\\ 0 & 1 & 0 & 0 & 0 & 0 & 0 & 0 & 0\\ 0 & 0 & 0 & 0 & 0 & 0 & 1 & 0 & 0\\ 0 & 0 & 0 & 0 & 0 & 0 & 0 & 1 & 0\\ 0 & 0 & 0 & 0 & 0 & 0 & 0 & 0 & 1 \end{array}\right]. \end{array}$$

Then


$$\begin{array}{c} \tilde{A}=\Pi A\Pi^{-1} \end{array}$$

has the block lower triangular form


$$\begin{array}{c} \tilde{A}=\left[\begin{array}{cc} A_{11} & 0\\ A_{21} & A_{22} \end{array}\right], \end{array}$$

where


$$\begin{array}{c} A_{11}=\left[\begin{array}{cccc} -\eta -\mu_{h}-\epsilon_{1} & 0 & 0 & 0\\ \eta & -\epsilon_{2}-\mu_{h} & 0 & 0\\ \epsilon_{1} & \epsilon_{2} & -\gamma_{1}-\mu_{h} & 0\\ 0 & 0 & q\gamma_{1} & -\gamma_{2}-\mu_{h} \end{array}\right], \end{array}$$

$$\begin{array}{c} A_{21}=\left[\begin{array}{cccc} 0 & 0 & 0 & 0\\ 0 & 0 & 0 & 0\\ 0 & 0 & \left(1-q\right)\gamma_{1} & \gamma_{2}\\ 0 & 0 & -\frac{\beta_{3}\Lambda_{v}}{u_{2}+\mu_{v}} & -\frac{\beta_{3}\Lambda_{v}}{u_{2}+\mu_{v}}\\ 0 & 0 & \frac{\beta_{3}\Lambda_{v}}{u_{2}+\mu_{v}} & \frac{\beta_{3}\Lambda_{v}}{u_{2}+\mu_{v}} \end{array}\right], \end{array}$$

and


$$\begin{array}{c} A_{22}=\left[\begin{array}{ccccc} -u_{1}-\mu_{h} & \delta & \omega & 0 & j_{1}\\ u_{1} & -\delta -\mu_{h} & 0 & 0 & -\frac{\left(1-p\right)\beta_{2}\Lambda_{h}u_{1}}{\mu_{h}\left(\delta +\mu_{h}+u_{1}\right)}-\frac{p\beta_{2}\Lambda_{h}u_{1}}{\mu_{h}\left(\delta +\mu_{h}+u_{1}\right)}\\ 0 & 0 & -\mu_{h}-\omega & 0 & 0\\ 0 & 0 & 0 & -u_{2}-\mu_{v} & 0\\ 0 & 0 & 0 & 0 & -u_{2}-\mu_{v} \end{array}\right]. \end{array}$$

Hence,


$$\begin{array}{c} \det\left(\lambda I-\tilde{A}\right)=\det\left(\lambda I-A_{11}\right)\det\left(\lambda I-A_{22}\right). \end{array}$$

Therefore, the characteristic polynomial of A factorizes into the product of the characteristic polynomials of *A*_11_ and *A*_22_. In particular, since *A*_22_ is upper triangular up to the last-column coupling, its explicit eigenvalues are


$$\begin{array}{c} \lambda_{1}=0,\;\lambda_{2}=-\mu_{h},\;\lambda_{3}=-u_{2}-\mu_{v},\;\lambda_{4}=-\mu_{h}-\omega ,\\ {\;\lambda }_{5}=-\delta -\mu_{h}-u_{1}, \end{array}$$

while the remaining four eigenvalues are obtained from


$$\begin{array}{c} \det\left(\lambda I-A_{11}\right)=0, \end{array}$$

which we confirmed through a numerical experiment to always have a negative real part. Since we have a simple zero eigenvalue of A, and the other eigenvalues have a negative real part, we continue our calculation by determining the right and left eigenvectors of A related to the zero eigenvalue. From direct calculation, the right eigenvector are given by $w={\left[w_{1},w_{2},\ldots ,w_{9}\right]}^{T}$ where:


$$\begin{array}{l} w_{1}=\frac{w_{11}}{{\left(\delta +\mu_{h}+u_{1}\right)}^{2}(\omega +\mu_{h})(q\gamma_{1}+\gamma_{2}+\mu_{h})((p\epsilon_{2}+\epsilon_{1}(1-p))\mu_{h}+\epsilon_{2}(\epsilon_{1}+\eta ))\Lambda_{v}\mu_{h}\beta_{3}},\\ w_{2}=\frac{w_{21}}{{\left(\delta +\mu_{h}+u_{1}\right)}^{2}(\omega +\mu_{h})(q\gamma_{1}+\gamma_{2}+\mu_{h})((p\epsilon_{2}+\epsilon_{1}(1-p))\mu_{h}+\epsilon_{2}(\epsilon_{1}+\eta ))\Lambda_{v}\mu_{h}\beta_{3}},\\ w_{3}\;=\frac{{\left(u_{2}+\mu_{v}\right)}^{2}(\gamma_{2}+\mu_{h})(\gamma_{1}+\mu_{h})(\epsilon_{2}+\mu_{h})(1-p)}{\beta_{3}\Lambda_{v}((p\epsilon_{2}+\epsilon_{1}(1-p))\mu_{h}+\epsilon_{2}(\epsilon_{1}+\eta ))(q\gamma_{1}+\gamma_{2}+\mu_{h})},\\ w_{4}\;=\frac{{\left(u_{2}+\mu_{v}\right)}^{2}(\gamma_{2}+\mu_{h})(\gamma_{1}+\mu_{h})(p\epsilon_{1}+p\mu_{h}+\eta )}{\beta_{3}\Lambda_{v}((p\epsilon_{2}+\epsilon_{1}(1-p))\mu_{h}+\epsilon_{2}(\epsilon_{1}+\eta ))(q\gamma_{1}+\gamma_{2}+\mu_{h})},\\ w_{5}\;=\frac{{\left(u_{2}+\mu_{v}\right)}^{2}(\gamma_{2}+\mu_{h})}{(q\gamma_{1}+\gamma_{2}+\mu_{h})\Lambda_{v}\beta_{3}},\\ w_{6}\;=\frac{q\gamma_{1}{\left(u_{2}+\mu_{v}\right)}^{2}}{(q\gamma_{1}+\gamma_{2}+\mu_{h})\Lambda_{v}\beta_{3}},\\ w_{7}\;=\frac{{\left(u_{2}+\mu_{v}\right)}^{2}\gamma_{1}((1-q)\mu_{h}+\gamma_{2})}{\Lambda_{v}\beta_{3}(\omega +\mu_{h})(q\gamma_{1}+\gamma_{2}+\mu_{h})},\\ w_{8}\;=-1,\\ w_{9}\;=1, \end{array}$$

while the left eigenvector *v* = [*v*_1_, *v*_2_, …, *v*_9_] with:


$$\begin{array}{cc} v_{1} & =0,\;v_{2}=0,\\ v_{3} & =\frac{\left(\mu_{h}\epsilon_{1}+\epsilon_{2}\left(\epsilon_{1}+\eta \right)\right)\beta_{3}\Lambda_{v}\left(q\gamma_{1}+\gamma_{2}+\mu_{h}\right)}{\left(u_{2}+\mu_{v}\right)\left(\gamma_{2}+\mu_{h}\right)\left(\gamma_{1}+\mu_{h}\right)\left(\epsilon_{2}+\mu_{h}\right)\left(\eta +\mu_{h}+\epsilon_{1}\right)},\\ v_{4} & =\frac{\Lambda_{v}\beta_{3}\left(q\gamma_{1}+\gamma_{2}+\mu_{h}\right)\epsilon_{2}}{\left(u_{2}+\mu_{v}\right)\left(\gamma_{2}+\mu_{h}\right)\left(\gamma_{1}+\mu_{h}\right)\left(\epsilon_{2}+\mu_{h}\right)},\\ v_{5} & =\frac{\Lambda_{v}\beta_{3}\left(q\gamma_{1}+\gamma_{2}+\mu_{h}\right)}{\left(u_{2}+\mu_{v}\right)\left(\gamma_{2}+\mu_{h}\right)\left(\gamma_{1}+\mu_{h}\right)},\\ v_{6} & =\frac{\Lambda_{v}\beta_{3}}{\left(u_{2}+\mu_{v}\right)\left(\gamma_{2}+\mu_{h}\right)},\\ v_{7} & =0,\;v_{8}=0,\;v_{9}=0. \end{array}$$

Based on the right and left eigenvectors associated with the simple zero eigenvalue of the Jacobian matrix at the disease-free equilibrium above, the coefficients (52) are given by


$$\begin{array}{c} A=\overset{9}{\underset{k,i,j=1}{\sum }}v_{k}w_{i}w_{j}\frac{\partial^{2}g_{k}}{\partial x_{i}\partial x_{j}}\left(0,0\right),\;B=\overset{9}{\underset{k,i=1}{\sum }}v_{k}w_{i}\frac{\partial^{2}g_{k}}{\partial x_{i}\partial \beta_{1}}\left(0,0\right). \end{array}$$

After direct calculation, we obtain the explicit expressions for A and B as follows:


$$\begin{array}{c} A=\sum_{k,i,j=1}^{9}v_{k}w_{i}w_{j}\frac{\partial^{2}g_{k}}{\partial x_{i}\partial x_{j}}\left(0,0\right)\\ =v_{1}w_{1}w_{1}\frac{\partial^{2}g_{1}}{\partial x_{1}\partial x_{1}}\left(0,0\right)+v_{1}w_{1}w_{2}\frac{\partial^{2}g_{1}}{\partial x_{1}\partial x_{2}}\left(0,0\right)+\cdots \\ +v_{9}w_{9}w_{9}\frac{\partial^{2}g_{9}}{\partial x_{9}\partial x_{9}}\left(0,0\right)\\ =\frac{-2\left(a_{8}\mu_{h}^{8}+a_{7}\mu_{h}^{7}+a_{6}\mu_{h}^{6}+a_{5}\mu_{h}^{5}+a_{4}\mu_{h}^{4}+a_{3}\mu_{h}^{3}+a_{2}\mu_{h}^{2}+a_{1}\mu_{h}+a_{0}\right)}{\Lambda_{v}\mu_{h}\left(u_{2}+\mu_{v}\right)\left(\eta +\mu_{h}+\epsilon_{1}\right)\left(\gamma_{2}+\mu_{h}\right)\left(\epsilon_{2}+\mu_{h}\right)\left(\omega +\mu_{h}\right){\left(\delta +\mu_{h}+u_{1}\right)}^{2}\left(\gamma_{1}+\mu_{h}\right)}, \end{array}$$

and


$$\begin{array}{c} B=\sum_{k,i=1}^{9}v_{k}w_{i}\frac{\partial^{2}g_{k}}{\partial x_{i}\partial \beta_{1}}\left(0,0\right)\\ =v_{1}w_{1}\frac{\partial^{2}g_{1}}{\partial x_{1}\partial \beta_{1}}\left(0,0\right)+v_{1}w_{1}\frac{\partial^{2}g_{1}}{\partial x_{2}\partial \beta_{1}}\left(0,0\right)+\cdots +v_{9}w_{9}\frac{\partial^{2}g_{9}}{\partial x_{9}\partial \beta_{1}}\left(0,0\right)\\ =\frac{\left(\mu_{h}\epsilon_{1}+\epsilon_{2}\left(\epsilon_{1}+\eta \right)\right)\beta_{3}\Lambda_{v}\left(q\gamma_{1}+\gamma_{2}+\mu_{h}\right)\left(1-p\right)\left(\delta +\mu_{h}\right)\Lambda_{h}}{\left(\eta +\mu_{h}+\epsilon_{1}\right)\left(\epsilon_{2}+\mu_{h}\right)\left(\gamma_{1}+\mu_{h}\right)\left(\gamma_{2}+\mu_{h}\right)\left(u_{2}+\mu_{v}\right)\mu_{h}\left(\delta +\mu_{h}+u_{1}\right)}\\ \;+\frac{\left(q\gamma_{1}+\gamma_{2}+\mu_{h}\right)\Lambda_{v}\beta_{3}\epsilon_{2}p\left(\delta +\mu_{h}\right)\Lambda_{h}}{\left(u_{2}+\mu_{v}\right)\left(\gamma_{2}+\mu_{h}\right)\left(\gamma_{1}+\mu_{h}\right)\left(\epsilon_{2}+\mu_{h}\right)\mu_{h}\left(\delta +\mu_{h}+u_{1}\right)}. \end{array}$$

Since all parameters are positive, the denominator of A is always positive. Furthermore, the numerator of A is preceded by a negative sign, while all coefficients in the polynomial are positive (which were also too long to be shown in this article). Hence, we have A<0. Similarly, all terms in the expression of B are positive, and therefore B>0 always holds.

According to the center manifold theorem of Castillo-Chavez and Song, the conditions A<0 and B>0 imply that the system undergoes a forward transcritical bifurcation at $R_{0}=1$. Hence, the proof is complete.

Based on the above theorem, the endemic equilibrium bifurcates from the disease-free equilibrium only when $R_{0}$ passes through one from below. In particular, this confirms that the endemic equilibrium exists only for $R_{0}>1$, and whenever it exists, it will always be locally asymptotically stable for $R_{0}>1$ but close to one. For the general case of $R_{0}>1$, we will present a stability analysis and a numerical experiment using the continuation technique with *Matcont*.

#### 3.3.1 Remarks on the analytical results

Based on Theorems 1–3, we have shown that the basic reproduction number $R_{0}$ acts as the main threshold parameter which always determines the qualitative behavior of malaria transmission in the proposed model. When $R_{0}<1$, the malaria-free equilibrium is locally asymptotically stable, indicating that malaria transmission cannot be sustained and the disease may die out in the population. Conversely, when $R_{0}>1$, a unique endemic equilibrium exists, showing that malaria may persist in the population at a positive endemic level. Furthermore, the occurrence of a forward bifurcation at $R_{0}=1$ indicates that the transition between disease elimination and disease persistence follows the usual threshold behavior. Therefore, reducing $R_{0}$ below unity remains a fundamental target for malaria control. In the context of this study, this can be achieved by strengthening intervention strategies such as PfSPZ vaccination and indoor residual spraying (IRS), which are designed to reduce human–mosquito transmission and suppress malaria persistence. We will further discuss these facts in the sensitivity analysis in the next section.

## 4 Parameter estimation using incidence data from Keerom, Indonesia

**Figure 3:**
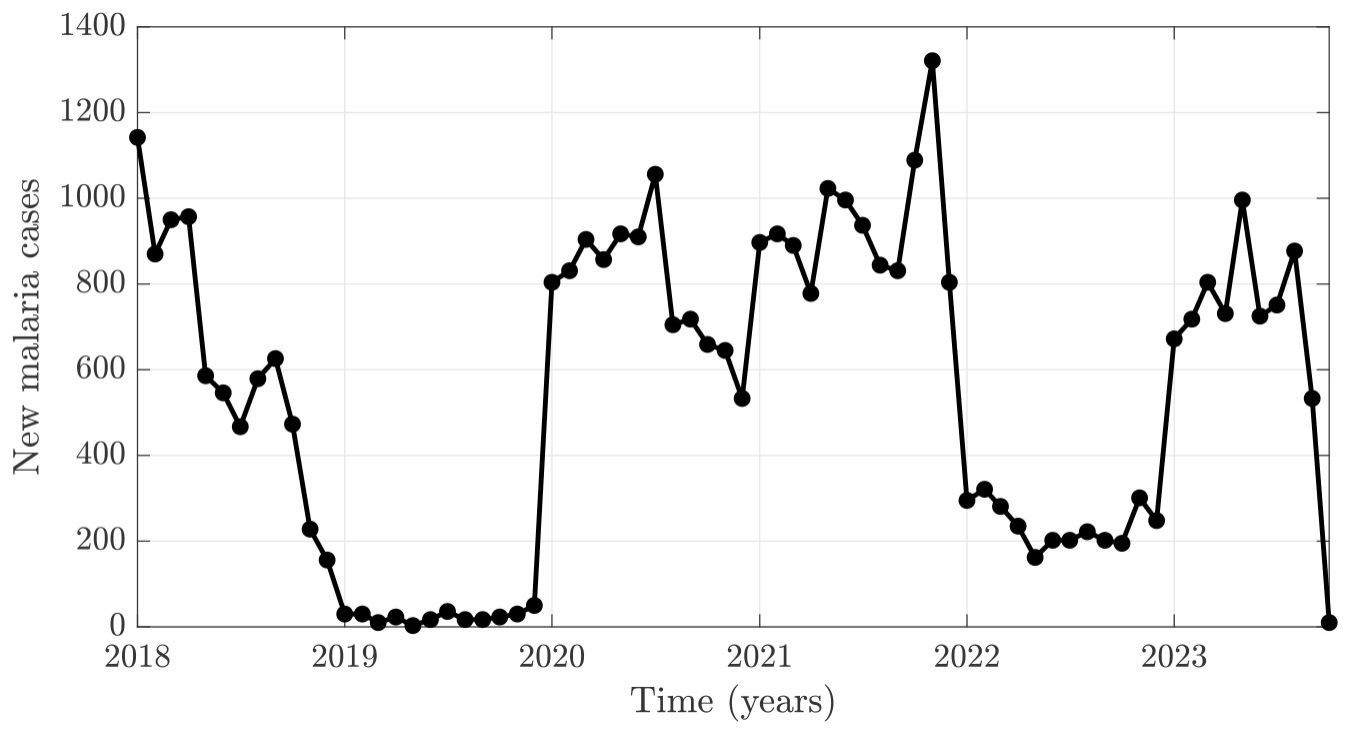
New monthly incidence data of Malaria in Keerom, from January 2018 to October 2023.

In this section, we discuss the incidence data used to estimate the model parameters in Equation 3. The malaria incidence data are taken from Keerom, a district in the province of Papua, Indonesia. Papua contributes more than 90% of the total malaria cases in Indonesia in 2023 (53). The total population of Keerom was 62,777 in 2023. The incidence data used in this study consist of monthly reported new malaria cases in Keerom, Indonesia, from January 2018 to October 2023. The data were obtained from Eijkman Research Center for Molecular Biology, National Research and Innovation Agency (BRIN). The data are presented in Figure 3.

To conduct the parameter estimation using data from Keerom, Indonesia, we consider that the available data represent the condition of the unvaccinated community in Keerom. Therefore, we use model (Equation 3) by neglecting the vaccinated compartment *V*, the average probability of successful transmission from mosquitoes to vaccinated humans β_2_, the vaccination rate *u*_1_, and the vaccine waning rate δ. Based on these assumptions, model (Equation 3) can be reduced as follows:


$$\begin{array}{l} \;\frac{dS}{dt}=\Lambda_{h}-\left(1-p\right)\beta_{1}SM_{2}-p\beta_{1}SM_{2}+\omega R-\mu_{h}S,\\ \;\frac{dD}{dt}=\left(1-p\right)\beta_{1}SM_{2}-\eta D-\epsilon_{1}D-\mu_{h}D,\\ \;\frac{dL}{dt}=p\beta_{1}SM_{2}+\eta D-\epsilon_{2}L-\mu_{h}L,\\ \;\frac{dI_{1}}{dt}=\epsilon_{1}D+\epsilon_{2}L-\left(1-q\right)\gamma_{1}I_{1}-q\gamma_{1}I_{1}-\mu_{h}I_{1},\\ \;\frac{dI_{2}}{dt}=q\gamma_{1}I_{1}-\gamma_{2}I_{2}-\mu_{h}I_{2},\\ \;\frac{dR}{dt}=\left(1-q\right)\gamma_{1}I_{1}+\gamma_{2}I_{2}-\omega R-\mu_{h}R,\\ \frac{dM_{1}}{dt}=\Lambda_{v}-\beta_{3}M_{1}\left(I_{1}+I_{2}\right)-\left(\mu_{v}+u_{2}\right)M_{2},\\ \frac{dM_{2}}{dt}=\beta_{3}M_{1}\left(I_{1}+I_{2}\right)-\left(\mu_{v}+u_{2}\right)M_{2}, \end{array}$$ (12)

The new cases of model Equation 12 are given by β_1_*SM*_2_. Hence, the accumulated cases (*c*) from our model in Equation 12, satisfy the following:


$$\begin{array}{c} \frac{dc}{dt}=\left(1-p\right)\beta_{1}SM_{2}+p\beta_{1}SM_{2}. \end{array}$$ (13)

**Figure 4:**
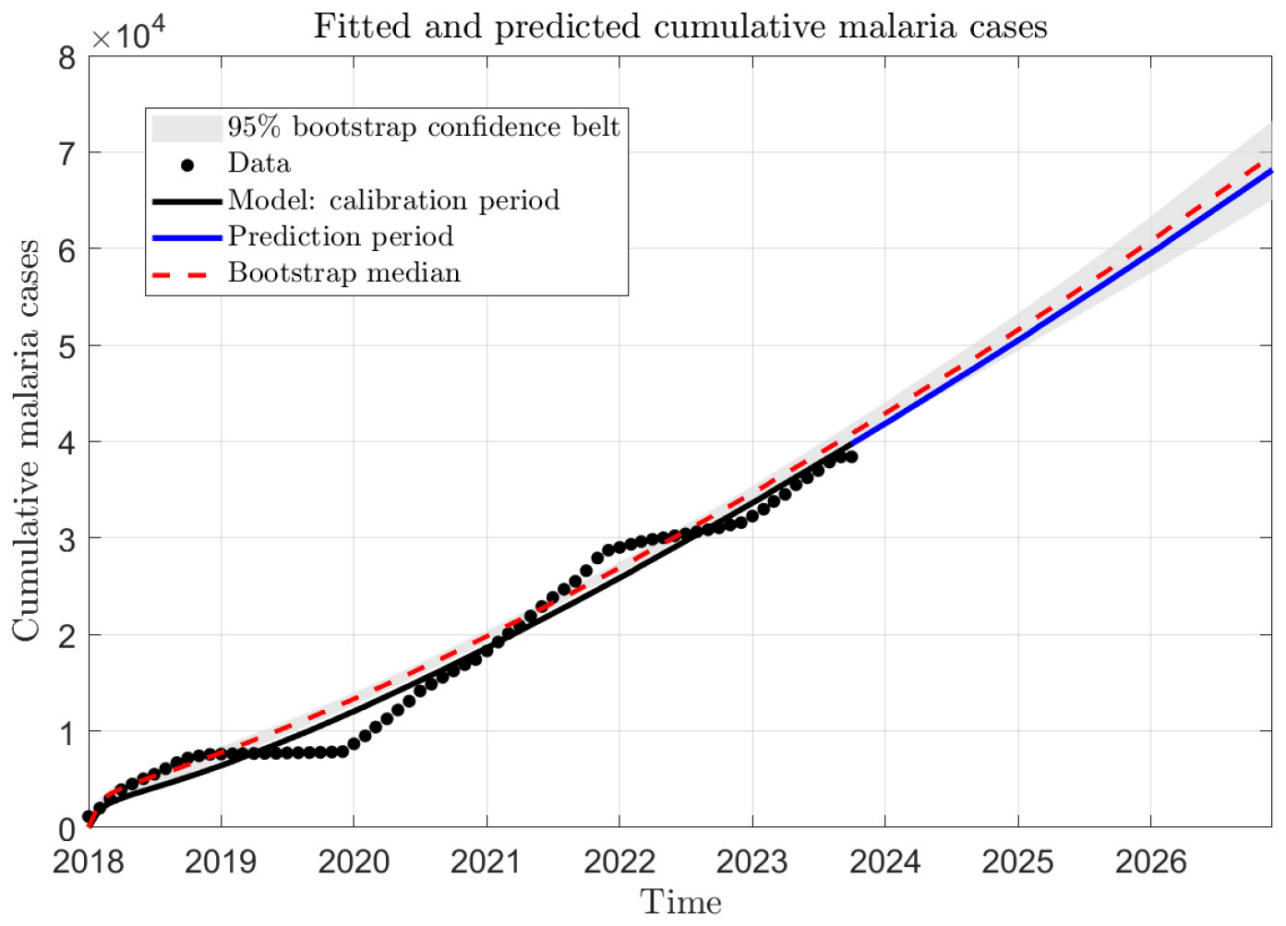
The parameter estimation results for fitting the data (dotted black curve), model output (blue curve), bootstrap median (red curve), and 95% bootstrap confidence belt (gray shade). The red dotted curve represents the forecasting of accumulated cases in Keerom from November 2023 to December 2026.

Solving the coupled set of differential equations in Equations 12, 13 gives us the accumulated cases of malaria incidence, which is shown by a dotted curve in Figure 4. We aim to fit our model output (*c*(*t*)) with the accumulated cases from Keerom, Papua. Mathematically, it can be translated as problem on minimizing the Euclidean distance between Malaria incidence data (denoted by *c*^data^) and the solution of *c*^model^ from system Equations 12, 13, using the best-fit parameter γ_1_, γ_2_, β_1_, β_3_, *p, q*, η, ϵ_1_, ϵ_2_, ω, *u*_2_ and best initial condition *S*(0), *D*(0), *L*(0), *I*_1_(0), *I*_2_(0), *R*(0), *M*_1_(0), and*M*_2_(0), while the other parameter that we do not fit were chosen based on literature as shown in Table 1. Hence, we define the following objective function


$$\begin{array}{c} C=\overset{70}{\underset{i=1}{\sum }}{\left(c_{i}^{\text{data}}-c_{i}^{\text{model}}\right)}^{2}, \end{array}$$

with *i* is the data point. We aim to seek the optimal parameters and initial conditions


$$\begin{array}{l} \Psi =\{\gamma_{1}^{*},\gamma_{2}^{*},\beta_{1}^{*},\beta_{3}^{*},p^{*},q^{*},\eta^{*},\epsilon_{1}^{*},\epsilon_{2}^{*},\omega^{*},u_{2}^{*},S^{*}(0),D^{*}(0),L^{*}(0),\\ \;I_{1}^{*}(0),I_{2}^{*}(0),R^{*}(0),M_{1}^{*}(0),M_{2}^{*}(0)\}, \end{array}$$

such that


$$\begin{array}{c} C\left(\text{Ψ}\right)=\underset{\text{Ψ}\in \Theta }{\min}C\left(\text{Ψ}\right), \end{array}$$

where Θ is the admissible value for the estimated parameters. The minimization problem was solved numerically using the MATLAB routine *fmincon*, which is suitable for constrained non-linear optimization problems. We start by providing an initial guess for all parameters we want to fit, which should fall within the possible interval values shown in Table 2. For each trial parameter set, the ODE system was solved numerically, the cumulative model output $c_{i}^{\text{model}}$ was evaluated at the observation times, and the corresponding cost function value was computed. The optimal parameter set was then selected as the one that minimized the least-squares discrepancy between the cumulative data and the cumulative model output. To quantify the uncertainty of the fitted trajectory, a residual bootstrap approach was applied. After obtaining the best-fit solution, residuals were computed as the difference between observed cumulative data and fitted cumulative model output. These residuals were resampled with replacement and added back to the fitted trajectory to create synthetic bootstrap data sets. For each bootstrap dataset, the parameter estimation was repeated using *fmincon* and the corresponding fitted and predicted model trajectories were stored. The empirical 2.5% and 97.5% percentiles of the bootstrap trajectories were then used to construct the 95% confidence belt around the model output. This bootstrap procedure provides an estimate of the uncertainty in the fitted and predicted cumulative malaria cases due to variability in the observed data.

**Table 2:** Lower and upper bounds of parameters that were used for fitting.

Parameters	Interval values	Sources
γ_1_	[0.3, 3]	(20, 54)
γ_2_	[0.3, 3]	(20, 54)
β_1_	$\left[\frac{0.062\times 0.5}{62777},\frac{0.062\times 1.5}{62777}\right]\times 30$	(20)
β_3_	$\left[\frac{0.048\times 0.5}{62777},\frac{0.048\times 1.5}{62777}\right]\times 30$	(20)
*p*	[0, 1]	Assumption
*q*	[0, 1]	Assumption
η	$\left[\frac{1}{\left(5\times 365\right)},\frac{1}{6}\right]\times 30$	(55, 56)
ϵ_1_	$\left[\frac{1}{10},\frac{1}{3}\right]\times 30$	(20)
ϵ_2_	$\left[\frac{1}{10},\frac{1}{3}\right]\times 30$	(20)
ω	[5.5 × 10^−4^, 110 × 10^−4^] × 30	(57)
*u*_2_	[0.15 × 0.5, 0.15 × 1.5] × 30	(58)

The results of our parameter estimation are given in Table 1, and the best-fit initial condition is given by


$$\begin{array}{l} S\left(0\right)=45671,\;D\left(0\right)=1699,\;L\left(0\right)=2606,\;I_{1}\left(0\right)=20277\\ I_{2}\left(0\right)=3155,R\left(0\right)=28830,\;M_{1}\left(0\right)=1.23\times 10^{5},\\ M_{2}\left(0\right)=4086. \end{array}$$

**Figure 5:**
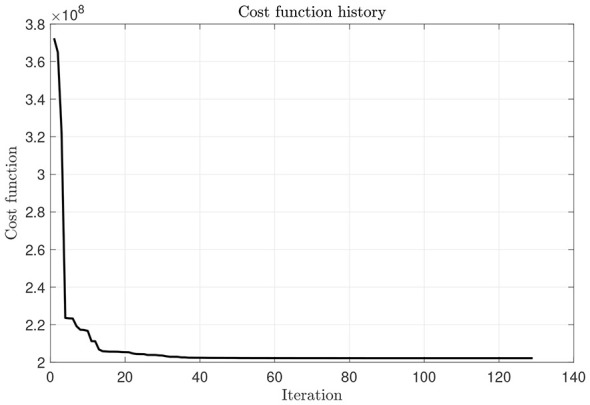
The value of objective function C for each iteration.

**Figure 6:**
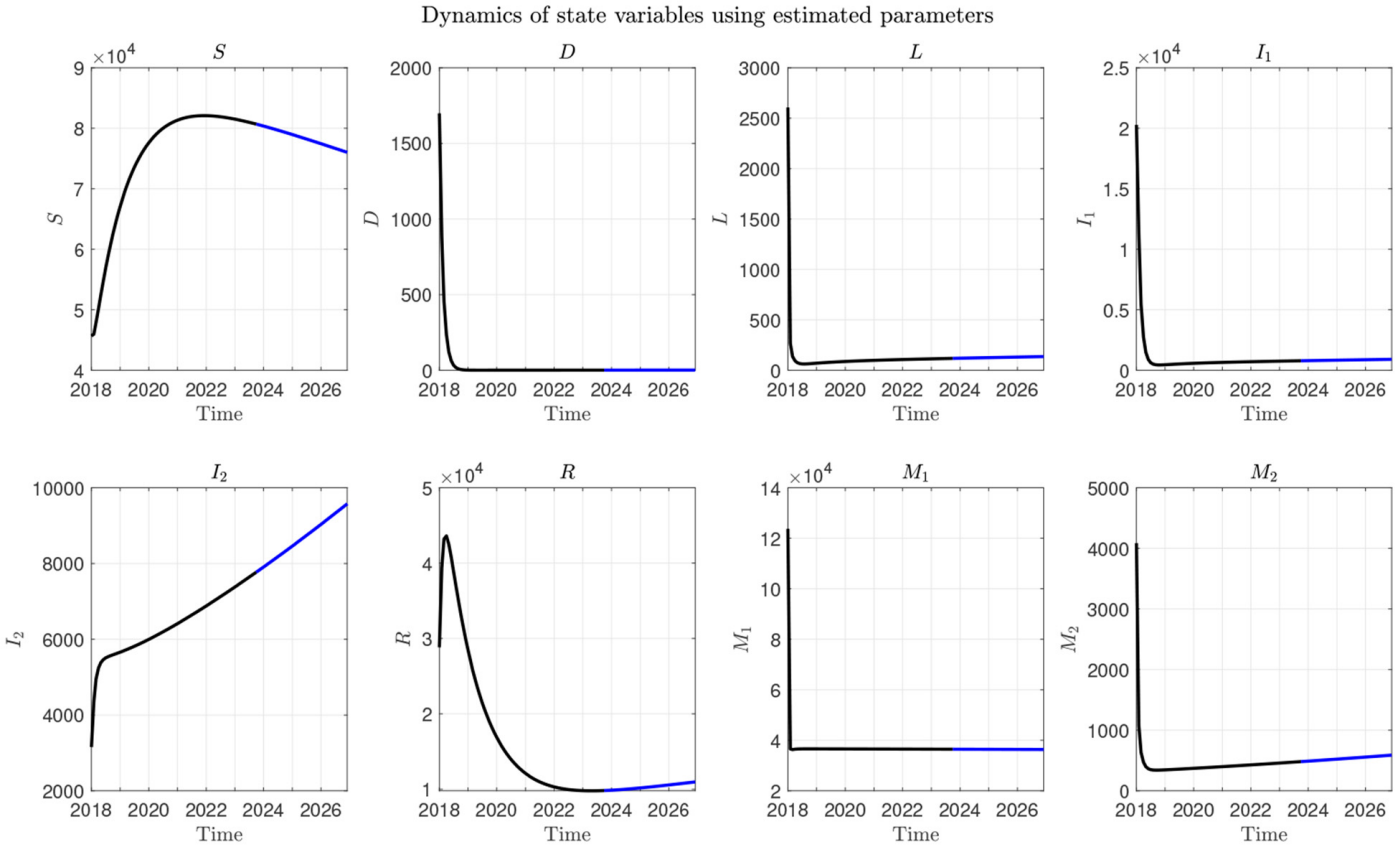
Model output of system Equation 12 using the estimated parameter values. The black curve represents the model output before November 2023, while the blue curve shows the extension up to December 2026.

From the parameter estimation illustrated in Figure 4, the cost function C for each iteration is shown in Figure 5. It can be seen that the cost function decreases monotonically, indicating that the estimate improves at each iteration until it reaches the minimum error allowed by the optimization procedure. Figure 4 also shows that the number of malaria cases still exhibits an increasing trend up to December 2026, as indicated by the blue curve. To complete the numerical experiment, we also present the dynamic of each variable in system Equation 12 until December 2026 as shown in Figure 6. Using the best-fit parameters in Table 1, we obtain $R_{0}=1.7$, indicating that malaria still has the potential to persist endemically in Keerom. Therefore, improvements in field interventions for malaria control in Keerom should be considered.

## 5 Sensitivity analysis

### 5.1 Global sensitivity analysis of $R_{0}$

**Figure 7:**
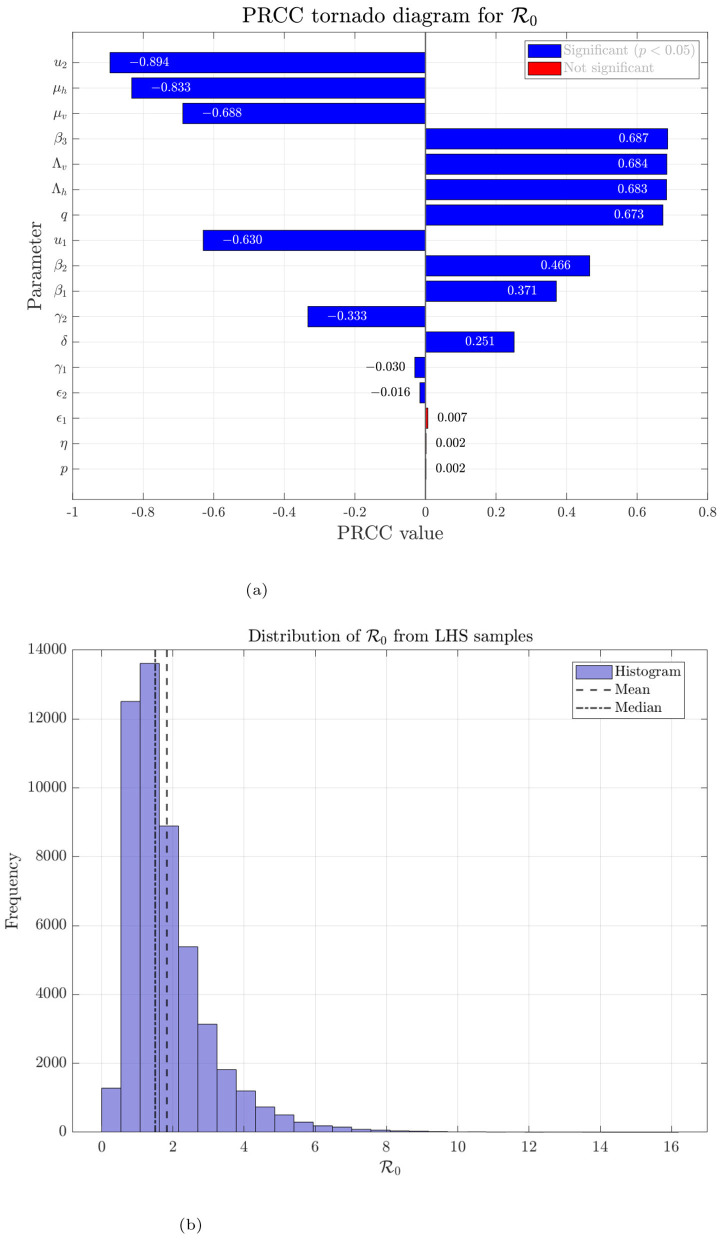
Numerical results for a global sensitivity of $R_{0}$. **(a)** PRCC analysis results of $R_{0}$. **(b)** Distribution of $R_{0}$ values from LHS sampling.

The sensitivity of the basic reproduction number $R_{0}$ to model parameters was explored using the Partial Rank Correlation Coefficient (PRCC) approach. This method is appropriate for non-linear models and allows evaluation of the monotonic relationship between input parameters and model output while accounting for the influence of other variables. Latin Hypercube Sampling (LHS) was used to create *N* = 50, 000 parameter sets within the permissible ranges ±50% of its best-fit parameters, and between 0 and 4 for *u*_1_ and *u*_2_. We calculate $R_{0}$ using the expression in Equation 5 value for each set of parameters sampled. The parameters sampled and the resulting values $R_{0}$ were rank-transformed before computing the PRCC values. Figure 7 shows the PRCC results, where Figure 7a shows the PRCC values and Figure 7b shows the distribution of $R_{0}$ values using the same sampling data that was used for Figure 7a. From 50,000 sampling points from eligible intervals for each parameter, we find that the mean of $R_{0}$ is 1.7 and the median is 1.4, which is close to the estimated $R_{0}$ from the best-fit parameter values for Kerom incidence data. Parameters with positive PRCC values increase the basic reproduction number, whereas negative values decrease it. We see that the control variables *u*_1_ and *u*_2_ show high PRCC values, with the vector control *u*_2_ being more significant in controlling $R_{0}$ than the vaccination *u*_1_. Both of these parameters show a negative PRCC value, indicating $R_{0}$ will always decrease whenever *u*_1_ or *u*_2_ increases. On the other hand, it is important to note that all infection parameters β_*i*_ for *i* = 1, 2, 3 have positive PRCC values, indicating that increasing these parameters will significantly increase $R_{0}$. It can be seen that the infection rate in mosquitoes (β_3_) is the most significant, followed by that in vaccinated individuals (β_2_), and lastly that in non-vaccinated individuals (β_1_). Furthermore, we can also see that the proportion of treated individuals who have failed in the first dose treatment (*q*) also has a significant PRCC value. Since it has a positive PRCC value, then more people failing in treatment will increase $R_{0}$ more significantly. Therefore, our experiment found that vector management, transmission-reduction tactics through vaccination, and reducing the probability of treatment failure have a significant impact on malaria dynamics, as indicated by sensitivity analysis of the basic reproduction number.

### 5.2 Numerical analysis of autonomous dynamics and $R_{0}$ contours

In this section, we conducted a sensitivity analysis of the control parameters and constructed a bifurcation diagram to validate our analytical results from the previous section. The parameter values in Table 1 were used to perform the numerical experiments, unless stated differently, and the initial condition is as follows:


$$\begin{array}{c} \left[S,V,D,L,I_{1},I_{2},R,M_{1},M_{2}\right]\\ =\left[60000,0,60,60,100,2557,6000,2\times 62727,100\right]. \end{array}$$

**Figure 8:**
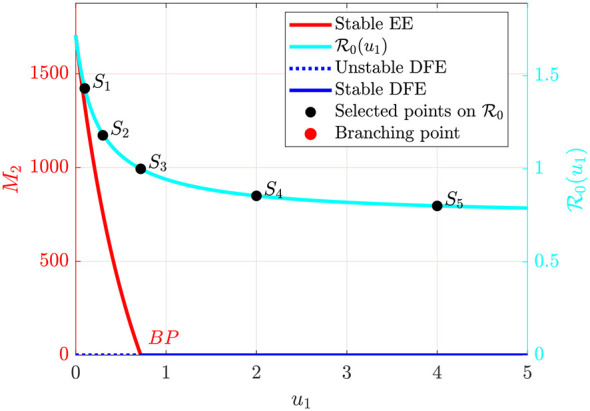
The bifurcation diagram of *M*_2_ with respect to *u*_1_ from model Equation 3. The solid red, blue, and cyan curves represent the stable endemic equilibrium, the stable malaria-free equilibrium, and the basic reproduction number as a function of *u*_1_. BP represents the bifurcation point $R_{0}=1$, while *S*_*i*_ at *i* = 1, 2, 3, 4, 5 denotes five sampling points for the autonomous simulation in Figure 10.

**Figure 9:**
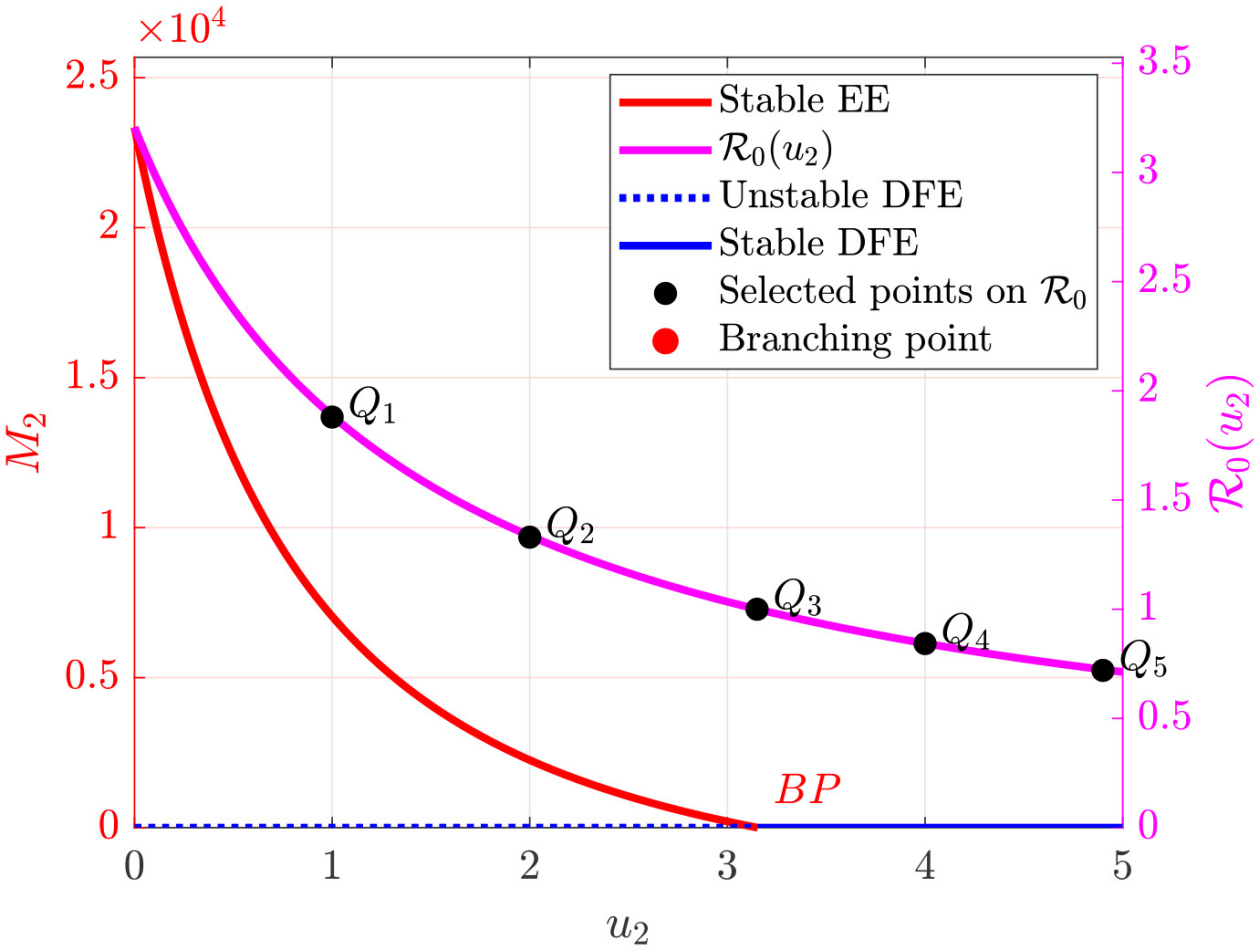
The bifurcation diagram of *M*_2_ with respect to *u*_2_ from model Equation 3. The solid red, blue, and cyan curves represent the stable endemic equilibrium, the stable malaria-free equilibrium, and the basic reproduction number as a function of *u*_2_. BP represents the bifurcation point $R_{0}=1$, while *Q*_*i*_ at *i* = 1, 2, 3, 4, 5 denotes five sampling points for the autonomous simulation in Figure 11.

**Figure 10:**
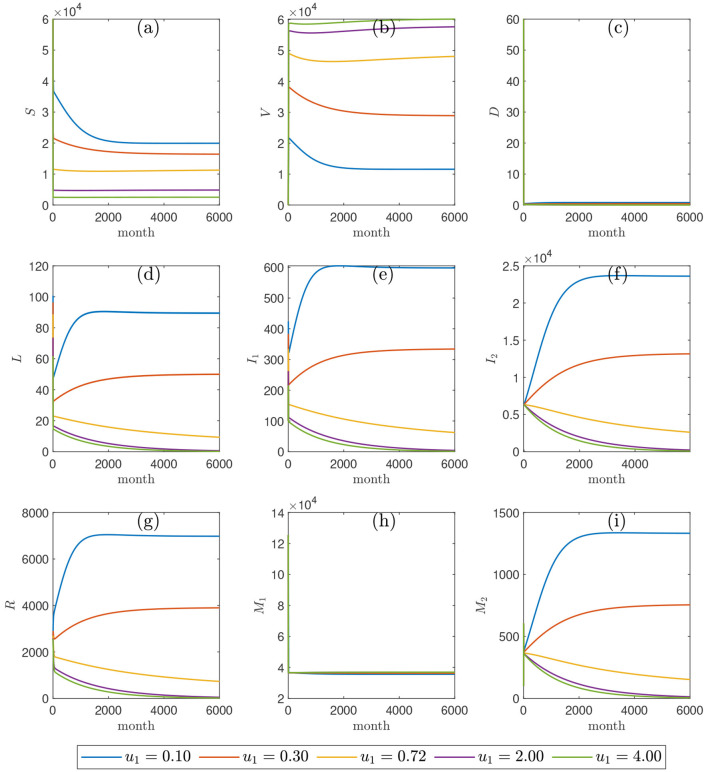
The autonomous simulation of System Equation 3 depends on the value of *u*_1_, which chosen based on 4 sampling points in Figure 8. **(a–g)** shows the dynamics of each human compartment, while **(h)** and **(i)** show the dynamics of the mosquito compartments.

We conducted the sensitivity analysis on the bifurcation diagram of System Equation 3 with respect to control parameters *u*_1_, and *u*_2_. The results are shown in Figure 8 for the bifurcation parameter *u*_1_ and Figure 9 for the bifurcation parameter *u*_2_. From Figure 8 we can see that the endemic equilibrium *M*_2_ is monotonically decreasing as *u*_1_ increases until it reaches the Bifurcation Point (BP) when $R_{0}=1\text{i.e.,}u_{1}=0.71$. For *u*_1_ <0.71, the malaria-free equilibrium is unstable, but the endemic equilibrium is stable. To illustrate the effects of *u*_1_ on the dynamic of *S, V, D, L, I*_1_, *I*_2_, *R, M*_1_, and *M*_2_, we particularly chose five sample points located at *S*_1_(*u*_1_ = 0.1), *S*_2_(*u*_1_ = 0.3), *S*_3_(*u*_1_ = 0.719), *S*_4_(*u*_1_ = 2), and *S*_5_(*u*_1_ = 4). The dynamics of *S, V, D, L, I*_1_, *I*_2_, *R, M*_1_, and *M*_2_ are plotted in Figures 10a–i. Increasing the value of *u*_1_ (rate of vaccination) increased the numbers of susceptible, dormant, latent, infected, treatment-failure, and recovered humans, as well as infected mosquitoes, thereby decreasing the numbers of vaccinated humans and susceptible mosquitoes.

**Figure 11:**
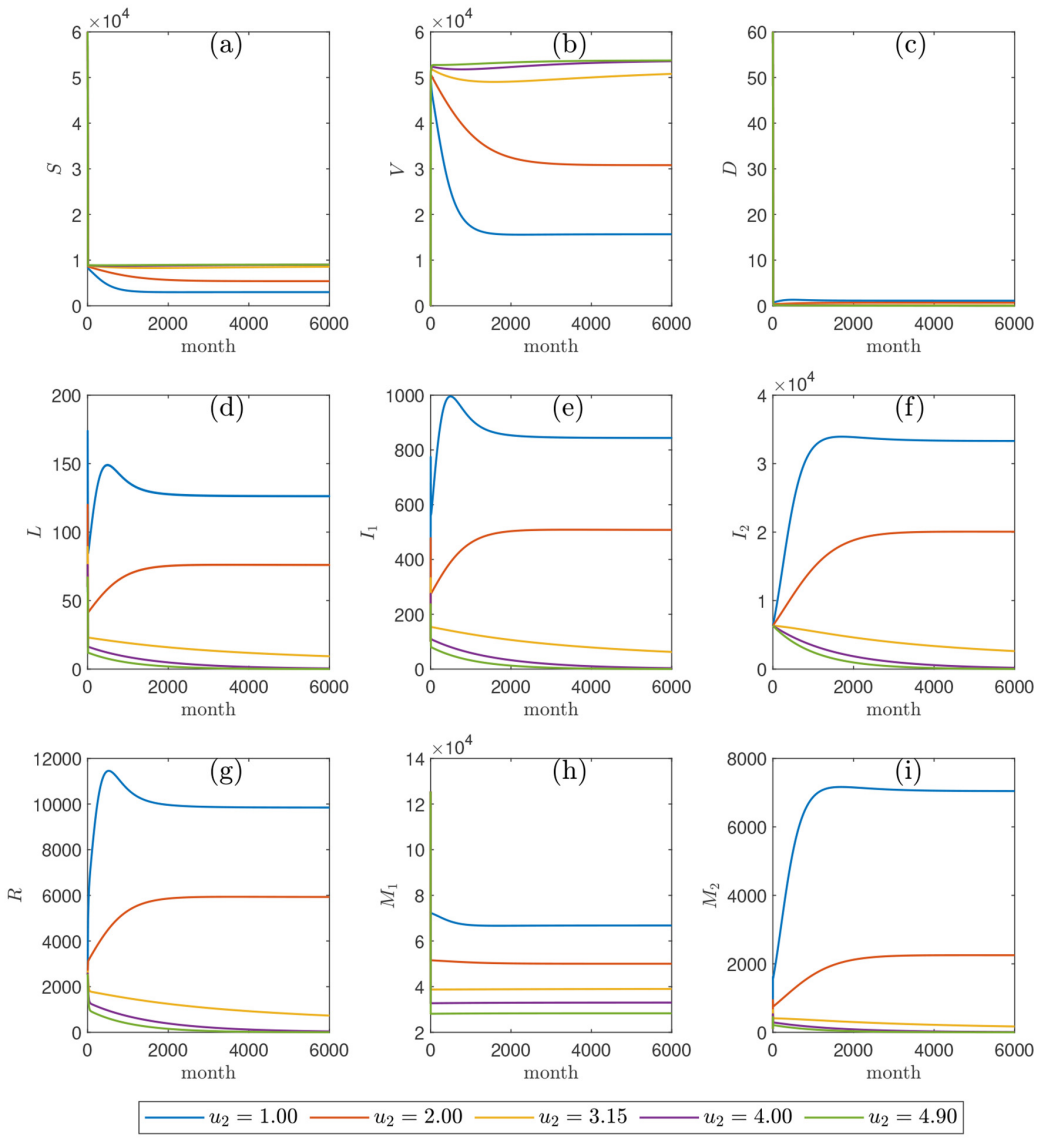
The autonomous simulation of system Equation 3 depends on the value of *u*_2_, which chosen based on four sampling points in Figure 9. **(a–g)** shows the dynamics of each human compartment, while **(h)** and **(i)** show the dynamics of the mosquito compartments.

**Figure 12:**
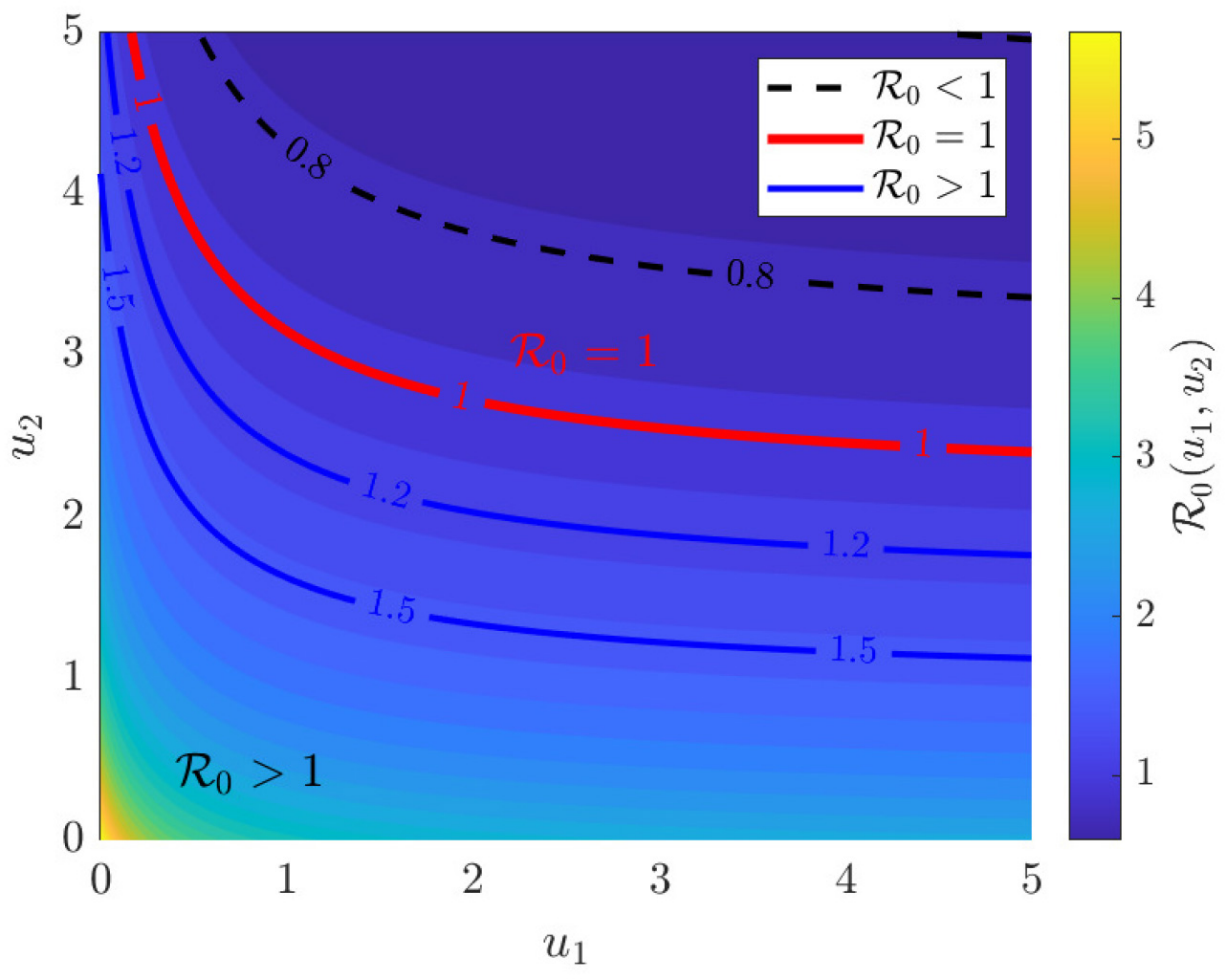
Contour plot of $R_{0}$ respect to *u*_1_ and *u*_2_.

For the next sensitivity analysis, we use the same parameter values and initial condition, except we set *u*_1_ = 0.001 and let *u*_2_ be the bifurcation parameter. From Figure 9 we can see that the endemic equilibrium *M*_2_ is monotonically decreasing as *u*_2_ increases until it reaches the Bifurcation Point (BP) when $R_{0}=1\text{i.e.,}u_{2}=3.15$. For *u*_2_ <3.15, the malaria-free equilibrium is unstable, but the malaria-endemic equilibrium is stable. To illustrate the effects of *u*_2_ on the dynamic of *S, V, D, L, I*_1_, *I*_2_, *R, M*_1_, and *M*_2_, we particularly chose four sample points located at *Q*_1_(*u*_2_ = 1), *Q*_2_(*u*_2_ = 2), *Q*_3_(*u*_2_ = 3.15), *Q*_4_(*u*_2_ = 4), and *Q*_5_(*u*_2_ = 4.9). The results of trajectories are plotted in Figures 11a–i. It is clear to see that a larger value of *u*_2_ increases *D, L, I*_1_, *I*_2_, *R, M*_1_, *M*_2_. As a closing to the local sensitivity analysis, we produce a contour plot of $R_{0}$ as a function of *u*_1_ and *u*_2_. The result is depicted in Figure 12. It can be seen that enlarging both controls can reduce $R_{0}$.

## 6 Optimal control model characterization and simulation

### 6.1 Existence of optimal control solution

Before we proceed to the characterization of our optimal control problem using Pontryagin's maximum principle, in this section, we first show the existence of a solution of our optimal control problem. The result is stated in the following theorem.

**Theorem 4 (Existence of an optimal control)**. Assume that all parameters in system Equation 1 are non-negative and that the state system admits a unique non-negative and bounded solution on [0, *T*] for every admissible control pair (*u*_1_, *u*_2_). Let the admissible control set be defined by


$$\begin{array}{l} U=\{(u_{1},u_{2})\in L^{\infty }(0,T{)}^{2}:0\leq u_{1}(t)\leq u_{1}^{\max},\;0\leq u_{2}(t)\leq u_{2}^{\max},\;\\ \;t\in [0,T]\}, \end{array}$$

where $u_{1}^{\max}$ and $u_{2}^{\max}$ are positive constants. If *c*_1_>0, *c*_2_>0, ω_1_≥0, and ω_2_≥0, then there exists an optimal control pair


$$\begin{array}{c} \left(u_{1}^{*},u_{2}^{*}\right)\in U \end{array}$$

such that


$$\begin{array}{c} J\left(u_{1}^{*},u_{2}^{*},X^{*}\right)=\underset{\left(u_{1},u_{2}\right)\in U}{\min}J\left(u_{1},u_{2},X\right), \end{array}$$

where X^*^ is the state solution of system Equation 1 corresponding to $\left(u_{1}^{*},u_{2}^{*}\right)$.

Proof. Please see Appendix B for the complete proof.

### 6.2 Optimal control characterization

Consider the optimal control model given in Equation 1, which incorporates two types of control variables, namely the vaccination rate *u*_1_(*t*) and the IRS rate *u*_2_(*t*). We aim to minimize the number of infected individuals in the human population (*I*_1_(*t*) and *I*_2_(*t*)) while keeping intervention costs as low as possible. This task can be mathematically described as an optimization problem, where we aim to minimize the cost function in Equation 2 subject to the admissible control set U.

The necessary condition for the optimal control problem is derived from Pontryagin's Maximum Principle (59). We define the Hamiltonian of our problem as follows:


$$\begin{array}{c} H=\omega_{1}I_{1}+\omega_{2}I_{2}+c_{1}u_{1}^{2}+c_{2}u_{2}^{2}+\overset{9}{\underset{i=1}{\sum }}\left(\lambda_{i}g_{i}\right), \end{array}$$ (14)

with *g*_*i*_ for *i* = 1, 2, …9 represent $\frac{dS}{dt},\frac{dV}{dt},\frac{dD}{dt},\frac{dL}{dt},\frac{dI_{1}}{dt},\frac{dI_{2}}{dt},\frac{dR}{dt},\frac{dM_{1}}{dt},$ and $\frac{dI_{2}}{dt}$, respectively. With the Pontryagin Maximum Principle (PMP), we have the following theorem.

**Theorem 5**. Let $\left(S^{*},V^{*},D^{*},L^{*},I_{1}^{*},I_{2}^{*},R^{*},M_{1}^{*},M_{2}^{*}\right)$ be the optimal state trajectories corresponding to an optimal control pair $\left(u_{1}^{*},u_{2}^{*}\right)\in U$. Then, there exist adjoint variables λ_*i*_(*t*), for *i* = 1, …, 9, satisfying the adjoint system


$$\begin{array}{l} \frac{d\lambda_{1}}{dt}=\left(\lambda_{1}-\lambda_{3}\right)\left(1-p\right)\beta_{1}M_{2}+\left(\lambda_{1}-\lambda_{4}\right)p\beta_{1}M_{2}\\ \;+\left(\lambda_{1}-\lambda_{2}\right)u_{1}+\lambda_{1}\mu_{h}, \end{array}$$ (15a)

$$\begin{array}{l} \frac{d\lambda_{2}}{dt}=\left(\lambda_{2}-\lambda_{1}\right)\delta +\left(\lambda_{2}-\lambda_{3}\right)\left(1-p\right)\beta_{2}M_{2}\\ \;+\left(\lambda_{2}-\lambda_{4}\right)p\beta_{2}M_{2}+\lambda_{2}\mu_{h}, \end{array}$$ (15b)

$$\begin{array}{c} \frac{d\lambda_{3}}{dt}=\left(\lambda_{3}-\lambda_{4}\right)\eta +\lambda_{3}\mu_{h}+\left(\lambda_{3}-\lambda_{5}\right)\epsilon_{1}, \end{array}$$ (15c)

$$\begin{array}{c} \frac{d\lambda_{4}}{dt}=\left(\lambda_{4}-\lambda_{5}\right)\epsilon_{2}+\lambda_{4}\mu_{h}, \end{array}$$ (15d)

$$\begin{array}{c} \frac{d\lambda_{5}}{dt}=-\omega_{1}+\left(\lambda_{5}-\lambda_{6}\right)q\gamma_{1}+\left(\lambda_{5}-\lambda_{7}\right)\left(1-q\right)\gamma_{1}\\ \;+\lambda_{5}\mu_{h}+\left(\lambda_{8}-\lambda_{9}\right)\beta_{3}M_{1}, \end{array}$$ (15e)

$$\begin{array}{c} \frac{d\lambda_{6}}{dt}=-\omega_{2}+\left(\lambda_{6}-\lambda_{7}\right)\gamma_{2}+\lambda_{6}\mu_{h}+\left(\lambda_{8}-\lambda_{9}\right)\beta_{3}M_{1}, \end{array}$$ (15f)

$$\begin{array}{c} \frac{d\lambda_{7}}{dt}=\left(\lambda_{7}-\lambda_{1}\right)\omega +\lambda_{7}\mu_{h}, \end{array}$$ (15g)

$$\begin{array}{c} \frac{d\lambda_{8}}{dt}=\left(\lambda_{8}-\lambda_{9}\right)\beta_{3}\left(I_{1}+I_{2}\right)+\lambda_{8}u_{2}+\lambda_{8}\mu_{v}, \end{array}$$ (15h)

$$\begin{array}{l} \frac{d\lambda_{9}}{dt}=\left(\lambda_{1}-\lambda_{3}\right)\left(1-p\right)\beta_{1}S+\left(\lambda_{1}-\lambda_{4}\right)p\beta_{1}S\\ \;+\left(\lambda_{2}-\lambda_{3}\right)\left(1-p\right)\beta_{2}V+\left(\lambda_{2}-\lambda_{4}\right)p\beta_{2}V\\ \;+\lambda_{9}\left(u_{2}+\mu_{v}\right). \end{array}$$ (15i)

with transversality condition λ_*i*_(*T*) = 0, and cost functional Equation 2. Furthermore, the optimal controls $u_{1}^{*}\left(t\right)$ and $u_{2}^{*}\left(t\right)$ are characterized by the minimization of the Hamiltonian almost everywhere on [0, *T*]. In particular, the optimal controls satisfy


$$\begin{array}{ccc} u_{1}^{*} & = & \min\left\{\max\left\{0,\frac{S\left(\lambda_{1}-\lambda_{2}\right)}{2c_{1}}\right\},u_{1,\max}\right\},\\ u_{2}^{*} & = & \min\left\{\max\left\{0,\frac{\lambda_{8}M_{1}+\lambda_{9}M_{2}}{2c_{2}}\right\},u_{2,\max}\right\}. \end{array}$$

Proof. The existence of an optimal control pair follows from the convexity of the integrand in Equation 2 with respect to the control variables and the fact that the admissible control set U is non-empty, closed, bounded, and convex. Furthermore, the state system Equation 1 is bounded in Ω.

The adjoint system Equation 15 derived from calculating the minus partial derivative of H respect to each state variable, i.e.,


$$\begin{array}{l} \frac{d\lambda_{1}}{dt}=-\frac{\partial H}{\partial S},\;\frac{d\lambda_{2}}{dt}=-\frac{\partial H}{\partial V},\;\frac{d\lambda_{3}}{dt}=-\frac{\partial D}{\partial S},\;\frac{d\lambda_{4}}{dt}=-\frac{\partial H}{\partial L},\\ \frac{d\lambda_{5}}{dt}=-\frac{\partial H}{\partial I_{1}},\;\frac{d\lambda_{6}}{dt}=-\frac{\partial H}{\partial I_{2}},\;\frac{d\lambda_{7}}{dt}=-\frac{\partial H}{\partial R},\;\frac{d\lambda_{8}}{dt}=-\frac{\partial H}{\partial M_{1}},\\ \frac{d\lambda_{9}}{dt}=-\frac{\partial H}{\partial M_{2}}. \end{array}$$

The transversality conditions λ_*i*_(*T*) = 0, *i* = 1, …, 9, follow from Pontryagin's Maximum Principle. Since the cost functional Equation 2 does not include any terminal cost and all state variables are free at the final time *T*, the adjoint variables must satisfy the natural transversality conditions λ_*i*_(*T*) = 0 for all *i*.

By applying the stationarity conditions of the Hamiltonian with respect to *u*_1_ and *u*_2_, the optimal controls are characterized by projecting the corresponding expressions onto their admissible bounds, namely between 0 and their respective upper bounds. Hence, the result follows for $u_{1}^{*}$ and $u_{2}^{*}$.

As a recap up to Theorem 5, our optimal control problem consists of the state system Equation 1, which represents our malaria model together with non-negative initial conditions, the cost function in Equation 2, the adjoint system Equation 15 equipped with the transversality condition λ_*i*_(*T*) = 0 for *i* = 1, 2, …, 9, and the optimal characterizations $u_{1}^{*}$ and $u_{2}^{*}$.

To solve this problem, we employ the forward–backward sweep method (60), which has also been implemented in Aldila et al. (61–63). The idea is to first provide an initial guess for the control variables *u*_1_(*t*) and *u*_2_(*t*) for all *t*∈[0, *T*]. With this initial guess, we solve the state system Equation 1 forward in time using the given initial conditions. Using the obtained state trajectories, we then solve the adjoint system Equation 15 backward in time, since it is equipped with terminal conditions. Once both the state and adjoint systems are obtained for *t*∈[0, *T*], the control variables are updated using the expressions for $u_{1}^{*}$ and $u_{2}^{*}$. This iterative procedure is repeated until the convergence criterion is satisfied, namely


$$\begin{array}{c} \underset{i=1,2}{\max}||u_{i}^{k+1}-u_{i}^{k}|{|}_{\infty }<\epsilon , \end{array}$$

where ϵ>0 is a prescribed tolerance, or until the maximum number of iterations is reached, whichever occurs first.

### 6.3 Numerical experiments on the optimal control problem

We consider three scenarios implementing control measures to eradicate malaria. The scenarios are (Scenario 1) Implementation of awareness program and usage of bed nets (*u*_1_, *u*_2_≠0); (Scenario 2) Implementation of awareness program only (*u*_1_ = 0, *u*_2_≠0); and (Scenario 3) Usage of bed nets only (*u*_1_≠0, *u*_2_ = 0). All parameters are given in Table 1.

#### 6.3.1 Scenario 1: *u*_1_≠0, *u*_2_≠0

**Figure 13:**
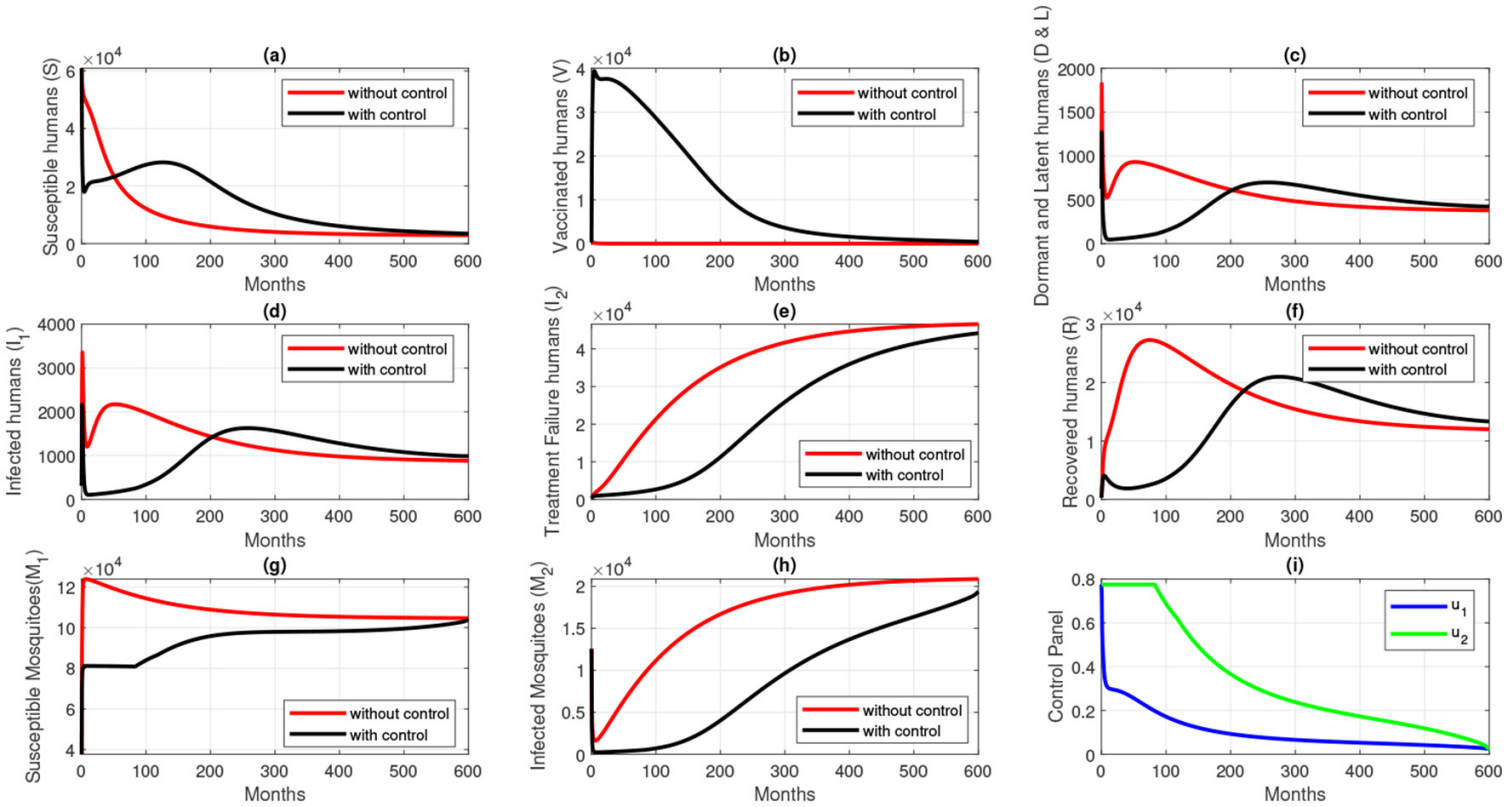
The dynamics of system Equation 1 under scenario 1, when *u*_1_≠0 and *u*_2_≠0. **(a–h)** show the dynamics of *S, V, D*+*L, I*_1_, *I*_2_, *R, M*_1_, and *M*_2_, respectively, while **(i)** shows the dynamics of the control interventions under Scenario 1.

For the first numerical experiment, we run our optimal control simulation with both control strategies implemented. Hence, we have vaccination and the IRS strategy coming into place together. The results is given in Figure 13 for 600 months simulation period, where we show the dynamic of *S*(*t*), *V*(*t*), *D*(*t*)+*L*(*t*), *I*_1_(*t*), *I*_2_(*t*), *R*(*t*), *M*_1_(*t*), *M*_2_(*t*) from panel (a) to (h), while the dynamic of controls are given in panel (i). The red and black curves represent the dynamics without and with control implemented, respectively.

Overall, the implementation of control strategies significantly alters disease dynamics by reducing the initial outbreak and introducing a delayed peak across several compartments. In panel (a), the susceptible population under control remains at a higher level for a longer period due to the reduced malaria infection pressure. The vaccinated population, as shown in panel (b), is extremely high at the beginning of the simulation period but decreases over time due to the reduced vaccination intensity. For the infection-related compartments in panels (c), (d), and (e), we observe that the outbreak is significantly reduced and delayed. The control profiles indicate that vaccination *u*_1_ is implemented intensively at the beginning and then rapidly decreases, whereas IRS *u*_2_ is sustained for a longer duration before gradually declining. These results suggest that the control strategies are effective in flattening and delaying the outbreak, although they may lead to a more prolonged persistence of the disease.

#### 6.3.2 Scenario 2: *u*_1_≠0, *u*_2_ = 0

**Figure 14:**
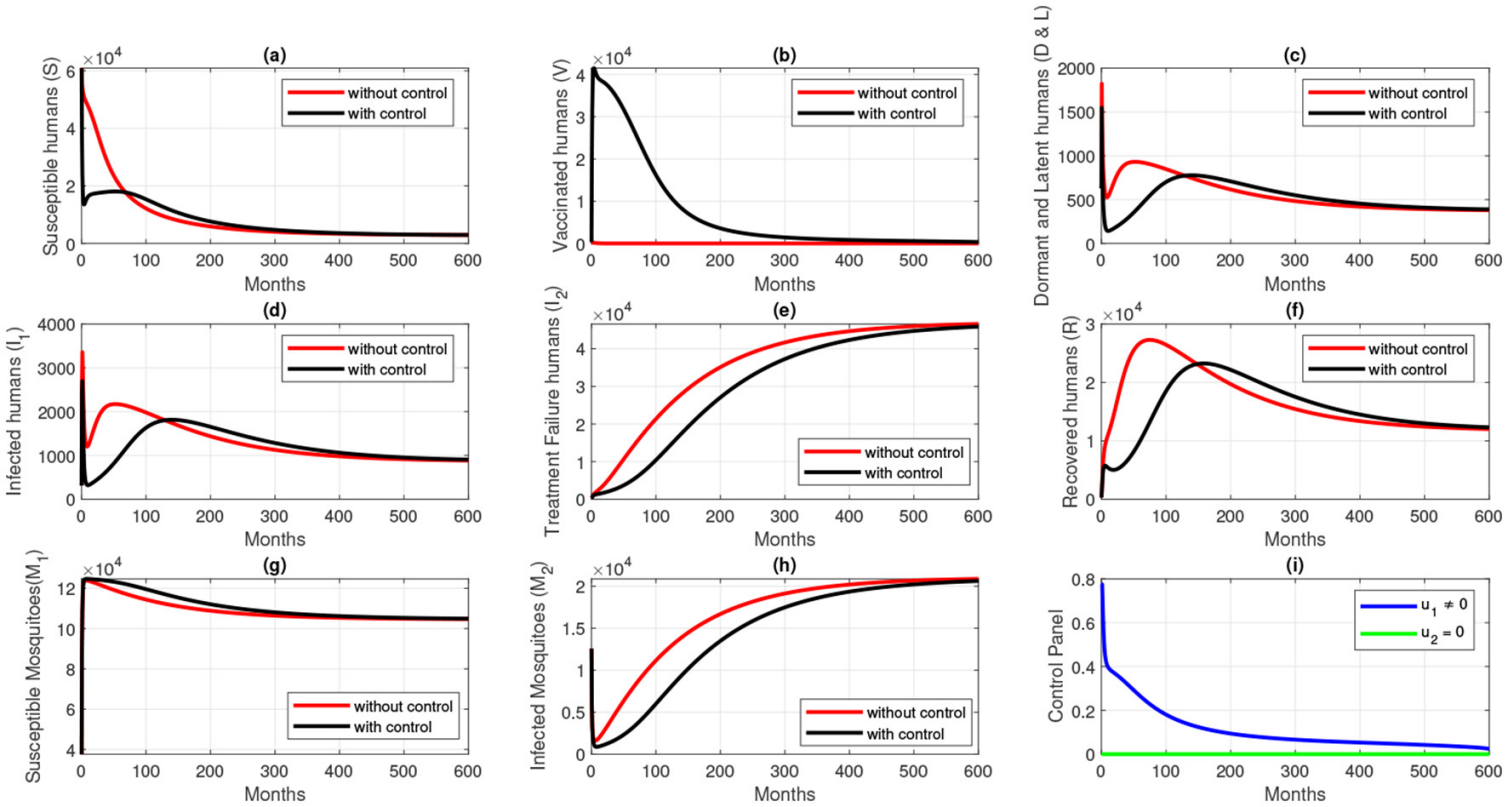
The dynamics of system Equation 1 under scenario 2, when *u*_1_≠0 and *u*_2_ = 0. **(a–h)** show the dynamics of *S, V, D*+*L, I*_1_, *I*_2_, *R, M*_1_, and *M*_2_, respectively, while **(i)** shows the dynamics of the control interventions under Scenario 2.

In the second scenario, we consider vaccination as the sole intervention, while IRS for vector control is absent. The results of this scenario are depicted in Figure 14. Compared to Scenario 1, the dynamics show a noticeably different impact of the control strategies. In this case, the reduction of the outbreak is less pronounced, and the control mainly affects the timing of the outbreak rather than reducing its magnitude.

In panel (a), although the vaccination strategy is still implemented, the susceptible population does not remain significantly higher than in the uncontrolled case, indicating that malaria transmission remains high due to the uncontrolled mosquito population. Similarly, the dynamics of infected individuals in panels (c), (d), and (e) do not show a significant reduction in the outbreak level. This suggests that the control strategy in this scenario is less effective in suppressing transmission intensity.

In the absence of the IRS strategy, vaccination intensity is slightly higher at the start of the simulation period than in Scenario 1, as shown in panel (i). Furthermore, even in the absence of the IRS, mosquito dynamics are still affected, albeit only slightly. Overall, compared to the previous scenario, the absence of IRS results in a weaker control outcome, highlighting the importance of combining multiple interventions to achieve a greater reduction in disease spread.

#### 6.3.3 Scenario 3: *u*_1_ = 0, *u*_2_≠0

**Figure 15:**
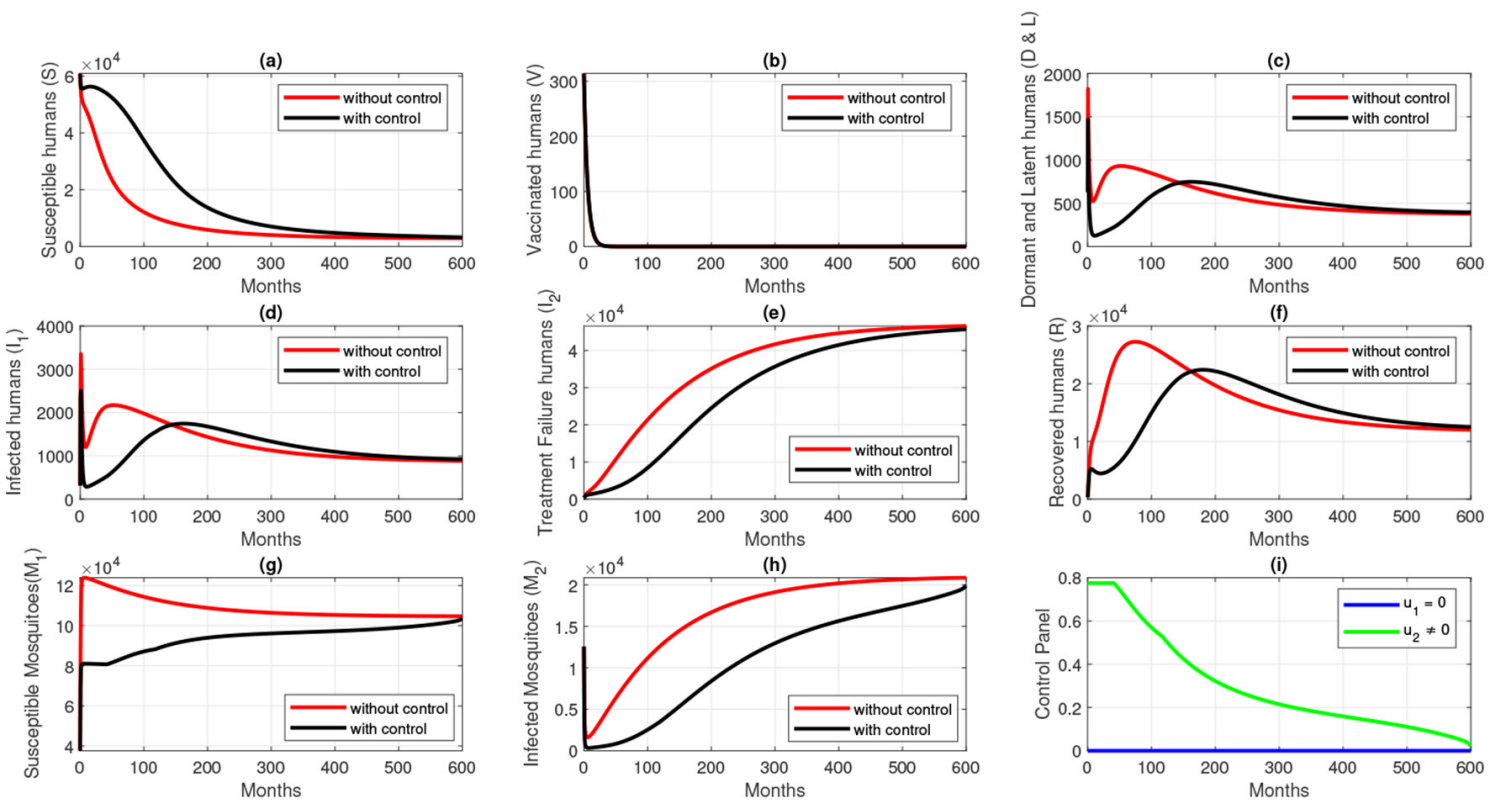
The dynamics of system Equation 1 under scenario 3, when *u*_1_ = 0 and *u*_2_≠0. **(a–h)** show the dynamics of *S, V, D*+*L, I*_1_, *I*_2_, *R, M*_1_, and *M*_2_, respectively, while **(i)** shows the dynamics of the control interventions under Scenario 3.

In the last scenario, we implement the IRS strategy as a single intervention, as shown in Figure 15, with vaccination absent. The dynamic of the IRS strategy can be seen in panel (i), where we can see that a slightly more intense IRS strategy should be implemented compared to Scenario 1.

Similar to Scenario 2, Scenario 3 shows a weaker overall reduction in malaria transmission than in Scenario 1, where both interventions are implemented. In panel (a), we can see that the susceptible population under control is still higher than in the uncontrolled case, but not as significantly as in Scenario 1. This result indicates that IRS alone can partially reduce infection pressure, but is less effective than the combined strategy. On the other hand, the vaccinated population strictly decreases due to the absence of vaccination, as shown in panel (b).

For the infected-related compartments in panels (c), (d), and (e), the outbreak is reduced and delayed compared to the uncontrolled case, but the reduction is not as strong as in Scenario 1. However, when compared to Scenario 2, the reduction is more pronounced, highlighting that IRS has a stronger impact on transmission than vaccination alone, particularly through its effect on the mosquito population. This is clearly reflected in panels (g) and (h), where both susceptible and infected mosquito populations are significantly reduced compared to both the uncontrolled case and Scenario 2. Overall, while the IRS alone is effective at suppressing vector dynamics and reducing transmission to some extent, it remains less effective than the combined strategy in Scenario 1.

### 6.4 Cost-effectiveness analysis

To determine the most cost-effective strategy among the three proposed strategies, we conduct a cost-effectiveness analysis. Cost-effectiveness analysis is an important tool in public health because it evaluates the efficiency of intervention strategies by comparing their implementation costs with their epidemiological benefits. In this study, three indicators are used, namely the Incremental Cost-Effectiveness Ratio (ICER), the Average Cost-Effectiveness Ratio (ACER), and the Infection Averted Ratio (IAR). The ICER compares the additional cost required to obtain an additional health benefit when moving from one strategy to another. It is useful for determining whether a more intensive strategy is worth the extra cost. The ACER calculates the average cost required to obtain one unit of health benefit for each strategy independently, thereby providing a simple measure of each intervention's average efficiency. Meanwhile, the IAR gives a direct epidemiological interpretation by measuring the number of infections averted per unit cost. Hence, these three indicators complement each other: ICER provides an incremental comparison between strategies, ACER evaluates the average efficiency of each strategy, and IAR highlights the practical benefit of each intervention in reducing malaria infection relative to its cost.

To calculate the ICER, ACER, and IAR, we first need to determine the total number of infections averted and the total cost for each scenario, as presented in Tables 3, 4. The total proportion of averted infections is calculated using the following formula:


$$\begin{array}{c} AI=\int_{0}^{T}\left[\overset{3}{\underset{i=1}{\sum }}\left(x_{i}\left(u_{j}\neq 0\right)-x_{i}\left(u_{j}=0\right)\right)\right]dt. \end{array}$$

**Table 3:** The Average Cost-Effectiveness Ratio (ACER) calculation for scenarios 1–3.

Scenarios	Controls	AI	CI	ACER
1	*u*_1_, *u*_2_	1.27 × 10^4^	0.339	2.67 × 10^−5^
2	*u*_1_	4.85 × 10^3^	0.225	4.65 × 10^−5^
3	*u*_2_	6.14 × 10^3^	0.139	2.27 × 10^−5^

AI, total averted infection; CI, total costs of the intervention.

**Table 4:** The incremental cost-effectiveness ratios (ICER's) increase in order based on scenarios 1–3.

Scenarios	Controls	AI	CI	ICER
2	*u*_1_	4.85 × 10^3^	0.225	4.65 × 10^−5^
3	*u*_2_	6.14 × 10^3^	0.139	−6.67 × 10^−5^
1	*u*_1_, *u*_2_	1.27 × 10^4^	0.339	3.04 × 10^−5^

AI, total averted infection; CI, total costs of the intervention.

for *j* = 1, 2, with *x*_1_, *x*_2_, and *x*_3_ represent *I*_1_, *I*_2_ and *M*_2_. Conversely, the total cost is given by equation $\overset{T}{\underset{0}{\int }}\left(\frac{\omega_{1}}{2}u_{1}^{2}+\frac{\omega_{2}}{2}u_{2}^{2}\right)dt$, while the total recovered individuals for each scenario is given by $R_{i}^{\text{tot}}=\overset{T}{\underset{0}{\int }}R_{i}\left(t\right)dt,$ for *i* = 1, 2, 3, . The results for all metrics are given in Tables 3–6.

**Table 5:** The incremental cost-effectiveness ratio's (ICER's) increasing order based on scenarios 2–3.

Scenarios	Controls	AI	CI	ICER
3	*u*_2_	6.14 × 10^3^	0.139	2.27 × 10^−5^
1	*u*_1_, *u*_2_	1.27 × 10^4^	0.339	3.04 × 10^−5^

AI, total averted infection; CI, total costs of the intervention.

**Table 6:** The Infection Averted Ratio (IAR) of scenarios 1–3.

Scenarios	Controls	AI	IR	IAR
1	*u*_1_, *u*_2_	1.27 × 10^4^	1.33 × 10^4^	0.95
2	*u*_1_	4.85 × 10^3^	1.55 × 10^4^	0.31
3	*u*_2_	6.14 × 10^3^	1.52 × 10^4^	0.4

AI, total averted infections; IR, total individuals recovered.

1. Average Cost-Effectiveness Ratio (ACER)The ACER represents the average cost per person averted from infection. Hence, the formula for ACER is given thus:

$$ACER_{\text{scenario}-i}=\frac{\text{Total cost for intervention with scenario }-i}{\text{Total of infection averted with scenario }-i}.$$

A smaller ACER indicates a better intervention strategy. The results of the ACER values for each strategy are in Table 3. According to Table 4, the best strategy based on the ACER index is the use of IRS (*u*_2_) as a single form intervention (Scenario 3), followed by scenarios 1 and 2, respectively. Therefore, we may say that Scenario 1 is the best intervention from an epidemiological perspective, while Scenario 3 is the most cost-effective strategy from an economic perspective. These findings suggest that IRS alone is the most efficient option when resources are limited, whereas a combined strategy is more suitable when the primary objective is to maximize disease reduction despite higher costs.

2. The Incremental Cost-Effectiveness Ratio (ICER)

The ICER reflects the cost-effectiveness of a scenario compared to an existing one. In general, a lower ICER suggests better cost-effectiveness, as it indicates that the scenario is achieved at a lower cost. The formula for calculating *ICER* is given below.


$$\begin{array}{c} ICER_{\text{scenario}-\left(i,J\right)}\\ =\frac{\text{Difference of costs between scenarios i \& j}}{\text{Difference of number of infections averted between scenarios i \& j}}. \end{array}$$

Based on the numerical simulation, we rank all strategies in increasing order of total infections averted in Tables 4, 5.

Notably, the ICER for Scenario 2 is larger than that of any other scenario. Hence, we can exclude the ICER for Scenario 2 from the next calculation. Subsequently, we compare the ICER between scenarios 3 and 1. The result of calculating ICER using the same method as before is demonstrated in Table 5.

Table 5 shows that ICER Scenario 1 > ICER Scenario 3, which means that Scenario 1 (vaccination program & IRS (*u*_1_, *u*_2_)) is more costly than Scenario 3 (IRS program (*u*_2_) implemented). Hence, we conclude that using IRS (*u*_2_) as a single intervention to reduce malaria transmission is the most cost-effective strategy compared with other scenarios. The ICER analysis is consistent with the ACER results, both indicating that Scenario 3 (IRS only) is the most cost-effective strategy. However, ICER provides a stronger decision framework by identifying Scenario 2 as a dominated strategy and confirming that the additional benefit of the combined intervention is associated with a higher incremental cost.

3. The Infection Averted Ratio (IAR)

The IAR is a measure of cost-effectiveness for preventing or reducing infections. A lower IAR would generally indicate better cost-effectiveness, as it implies that a relatively lower cost is associated with preventing or averting each infection. The formula is as follows.


$$IAR_{\text{scenario}-i}=\frac{\text{Total of infections averted with Scenario }-i}{\text{Total of individuals recovered with Scenario }-i}.$$

A higher IAR indicates a better interventional strategy. The IAR values for each strategy are visualized in Table 6. The IAR analysis provides a different perspective compared to the ACER and ICER results. While ACER and ICER identify Scenario 3 (IRS only) as the most cost-effective strategy, the IAR results indicate that Scenario 1 (combined intervention) performs best, achieving the highest ratio of infections averted per recovered individual.

## 7 Conclusion

In this article, we propose a novel mathematical model to explore the potential of the PfSPZ vaccine to control malaria transmission. With the implementation of this vaccine, the probability of successful malaria transmission can be significantly reduced. Furthermore, a vector control strategy using indoor residual spraying (IRS) is also incorporated as an additional intervention. Both interventions are included in our novel host–vector model, formulated as an optimal control problem, in which we aim to determine the most effective strategy to reduce the number of infected humans and mosquitoes while maintaining an optimal level of intervention effort. The model is formulated as a system of ordinary differential equations that accommodates seven distinct human subpopulations and two mosquito subpopulations. Unlike the authors in Aldila et al. (48), we specifically introduce dormant, latent, and treatment-failure compartments to provide a more realistic representation of malaria transmission dynamics.

Mathematical analysis of the model was conducted rigorously, including the proof of solution positivity, the derivation of the malaria-free and malaria-endemic equilibria and their stability, and the computation of the basic reproduction number. Our analysis reveals that the basic reproduction number serves as a threshold parameter for malaria persistence in the population. When the basic reproduction number is less than one, the malaria-free equilibrium is locally asymptotically stable, whereas when it exceeds one, a unique stable malaria-endemic equilibrium exists. This phenomenon is characterized by a transcritical bifurcation occurring at the critical point where the basic reproduction number equals one. The bifurcation analysis is carried out using a center manifold approach based on the Castillo–Song bifurcation theorem (52).

To make the model more realistic, we estimate our model parameters using novel incidence data from Kerom in Papua province, Indonesia. The incidence data were collected from January 2018 up to October 2023. Using this data, we estimated our parameter values and attempted to forecast the potential dynamics of accumulated cases in Kerom through April 2026. From our numerical experiment, we show that the accumulated cases still increase, indicating that malaria cases in Kerom may still arise. These results were supported by the calculation of the basic reproduction number using the best-fit parameter, which is 1.7, indicating a stable malaria-endemic equilibrium.

We acknowledge that our estimated parameter values are robust; hence, it is important to conduct the sensitivity analysis. To do this, we performed a global sensitivity analysis of the basic reproduction number using Partial Rank Correlation Coefficient (PRCC) combined with Latin Hypercube Sampling (LHS). Our analysis reveals that the vaccination strategy and vector control with IRS (*u*_1_ and *u*_2_, respectively), along with the transmission parameters β_1_, β_2_, and β_3_, play a dominant role in increasing the basic reproduction number $R_{0}$. This indicates that both transmission reduction and vector control strategies are crucial in mitigating malaria spread, with IRS-based vector control strategies being the more dominant feasible malaria control approach than vaccination.

For the final numerical experiment, we address the optimal control problem using the Pontryagin Maximum Principle (PMP) and solved it numerically using the forward–backward sweep method (60). Three scenarios were considered in this study, in which we examined all possible combinations of vaccination and IRS strategies. A cost-effectiveness analysis based on ICER, ACER, and IAR was conducted to identify the most cost-effective strategy. From the results, we observe that the IAR analysis offers a different perspective from the ACER and ICER results, highlighting the importance of considering multiple evaluation metrics when assessing intervention strategies. While ACER and ICER identify Scenario 3 (IRS only) as the most cost-effective strategy, the IAR results indicate that Scenario 1 (combined intervention) performs best, achieving the highest ratio of infections averted per recovered individual. This suggests that the combined strategy is more effective at reducing transmission than at slowing disease progression, even though it incurs a higher cost. These differences highlight that the choice of metric plays an important role in evaluating intervention strategies. ACER and ICER emphasize economic efficiency, favoring lower-cost strategies, whereas IAR emphasizes epidemiological effectiveness. Therefore, although IRS alone is the most cost-effective option, the combined intervention provides the greatest overall impact in reducing malaria transmission. This reinforces the trade-off between cost and effectiveness, where the optimal strategy depends on whether the priority is minimizing cost or maximizing health outcomes.

Overall, our research reveals the potential of the PfSPZ vaccine in controlling malaria transmission in endemic settings, particularly when implemented alongside vector control strategies such as IRS. The implementation of both strategies not only reduces outbreaks but also enhances the overall effectiveness of interventions in endemic settings. Implementation of the vaccine will lower the intensity of the IRS strategy, which, in many cases, has been reported to have a high potential to trigger mosquito resistance to insecticides. Although it has many advantages, this study still has several limitations. The model is deterministic and does not explicitly account for spatial heterogeneity, stochastic effects, human mobility, age structure, seasonal rainfall, or variation in mosquito species. Some parameters are obtained from the literature, while the monthly incidence data used for calibration are subject to reporting and data-sharing limitations. Furthermore, PfSPZ vaccination and IRS are represented as continuous control functions, although their implementation in the field may be discrete and constrained by logistical and behavioral factors. Therefore, the numerical and cost-effectiveness results should be interpreted as comparative insights into the potential impact of vaccination and IRS, rather than as direct operational recommendations. For future research, it would be necessary to extend the model by incorporating spatial heterogeneity, seasonal variability, and more detailed human behavioral responses toward vaccination and vector control measures.

## Data Availability

The raw data supporting the conclusions of this article will be made available by the authors, without undue reservation.
