# Supplementary material for: Optimal control and cost-effectiveness analysis of PfSPZ vaccination and indoor residual spraying in malaria transmission: a case study in Keerom
Source: Front Public Health. 2026 Jul 1;14:1872970. doi: 10.3389/fpubh.2026.1872970 (PMC13369308; doi:10.3389/fpubh.2026.1872970)
Supplement: Supplementary file 1 [file Supplementary_file_1.pdf]

Appendix: Optimal Control and Cost-Effectiveness Analysis of PfSPZ Vaccination and Indoor Residual Spraying in Malaria Transmission: A Case Study in Keerom

1 Proof of local stability of the MFE.

To apply the local stability criteria of the disease-free equilibrium of the epidemic model in [54], we need to verify that our model satisfies the sufficient conditions (A1)–(A5) in the mentioned reference. Following [54], we arrange the first m compartments to correspond to the infected compartments, namely D, L, I_1, I_2, M_2. The next compartments correspond to the non-infected compartments, namely, in sequential order, S, V, R, and M_1. Hence, we have $m = 1$ to 5 for the infected compartments, while $m = 6$ to 9 correspond to the non-infected compartments. Hence, let $x = (D, L, I_1, I_2, M_2, S, V, R, M_1)$. Next, we define the new infection terms ($\mathscr{F}$) and all other transitions ($\mathscr{V}$) as follows:

$$\mathscr{F}(x) = \begin{pmatrix} (1-p)M_2(\beta_1 S + \beta_2 V) \\ pM_2(\beta_1 S + \beta_2 V) \\ 0 \\ 0 \\ \beta_3 M_1(I_1 + I_2) \\ 0 \\ 0 \\ 0 \\ 0 \end{pmatrix}, \quad \mathscr{V}(x) = \begin{pmatrix} (\eta + \epsilon_1 + \mu_h)D \\ (\epsilon_2 + \mu_h)L - \eta D \\ (\gamma_1 + \mu_h)I_1 - \epsilon_1 D - \epsilon_2 L \\ (\gamma_2 + \mu_h)I_2 - q\gamma_1 I_1 \\ (\mu_v + u_2)M_2 \\ \beta_1 S M_2 + (u_1 + \mu_h)S - \Lambda_h - \delta V - \omega R \\ \beta_2 V M_2 + (\delta + \mu_h)V - u_1 S \\ (\omega + \mu_h)R - (1-q)\gamma_1 I_1 - \gamma_2 I_2 \\ (\mu_v + u_2)M_1 - \Lambda_v \end{pmatrix}. \tag{1}$$

Furthermore, we decompose $\mathscr{V}(x)$ as $\mathscr{V} = \mathscr{V}^-(x) - \mathscr{V}^+(x)$ as follows:

$$\mathscr{V}^-(x) = \begin{pmatrix} (\eta + \epsilon_1 + \mu_h)D \\ (\epsilon_2 + \mu_h)L \\ (\gamma_1 + \mu_h)I_1 \\ (\gamma_2 + \mu_h)I_2 \\ (\mu_v + u_2)M_2 \\ \beta_1 S M_2 + (u_1 + \mu_h)S \\ \beta_2 V M_2 + (\delta + \mu_h)V - u_1 S \\ (\omega + \mu_h)R \\ (\mu_v + u_2)M_1 + \beta_3 M_1(I_1 + I_2) \end{pmatrix}, \quad \mathscr{V}^+(x) = \begin{pmatrix} 0 \\ \eta D \\ \epsilon_1 D + \epsilon_2 L \\ q\gamma_1 I_1 \\ 0 \\ \Lambda_h + \delta V + \omega R \\ u_1 S \\ (1-q)\gamma_1 I_1 + \gamma_2 I_2 \\ \Lambda_v \end{pmatrix}. \tag{2}$$

With the above definition, we now proceed to the proof of axioms (A1) to (A5) in [54]. For condition (A1), it can be easily checked that for all $x \geq 0$, all $\mathscr{F}_i(x), \mathscr{V}_i^-(x)$, and $\mathscr{V}_i^+(x)$ are nonnegative. For condition (A2), we can easily check that whenever $x = 0$, then $\mathscr{V}_i^-(x) = 0$ always holds. For (A3), we note that for $i > 5$, we have $\mathscr{F}_i(x) = 0$ always holds. Next, let us denote X_s as the malaria-free equilibrium, which is $x_1 = x_2 = x_3 = x_4 = x_5 = 0$, and $x_i \geq 0$ for $i = 6, 7, 8, 9$ in X_s. Since substituting $x \in X_s$ gives $\mathscr{F}_i(x) = \mathscr{V}_i(x) = 0$ for $i = 1, 2, 3, 4, 5$, condition (A4) is also satisfied. By direct calculation, we have:

$$\mathscr{J}(X_s, \mathscr{F}(x) = 0) = \left[\begin{array}{c|c|c} A_{11} & A_{12} & A_{13} \\ \hline A_{21} & A_{22} & A_{23} \\ \hline A_{31} & A_{32} & A_{33} \end{array}\right], \tag{3}$$

where

$$A_{11} = \begin{bmatrix} -\eta-\epsilon_1-\mu_h & 0 & 0 \\ \eta & -\epsilon_2-\mu_h & 0 \\ \epsilon_1 & \epsilon_2 & -\gamma_1-\mu_h \end{bmatrix}, \quad A_{12} = \begin{bmatrix} 0 & 0 & 0 \\ 0 & 0 & 0 \\ 0 & 0 & 0 \end{bmatrix}, \quad A_{13} = \begin{bmatrix} 0 & 0 & 0 \\ 0 & 0 & 0 \\ 0 & 0 & 0 \end{bmatrix},$$

$$A_{21} = \begin{bmatrix} 0 & 0 & q\gamma_1 \\ 0 & 0 & 0 \\ 0 & 0 & 0 \end{bmatrix}, \quad A_{22} = \begin{bmatrix} -\gamma_2-\mu_h & 0 & 0 \\ 0 & -\mu_v-u_2 & 0 \\ 0 & 0 & -\mu_h-u_1 \end{bmatrix}, \quad A_{23} = \begin{bmatrix} 0 & 0 & 0 \\ 0 & 0 & 0 \\ \delta & \omega & 0 \end{bmatrix},$$

$$A_{31} = \begin{bmatrix} 0 & 0 & 0 \\ 0 & 0 & (1-q)\gamma_1 \\ 0 & 0 & 0 \end{bmatrix}, \quad A_{32} = \begin{bmatrix} u_1 & -\delta-\mu_h & 0 \\ 0 & 0 & -\omega-\mu_h \\ 0 & 0 & 0 \end{bmatrix}, \quad A_{33} = \begin{bmatrix} 0 & 0 & 0 \\ \gamma_2 & 0 & 0 \\ 0 & 0 & -\mu_v-u_2 \end{bmatrix}.$$

From direct calculation, the eigenvalues of the above matrix are $-(\eta+\epsilon_1+\mu_h)$, $-(\mu_v+u_2)$, $-(\mu_v+u_2)$, $-(\gamma_1+\mu_h)$, $-(\gamma_2+\mu_h)$, $-(\epsilon_2+\mu_h)$, $-\mu_h$, $-(\omega+\mu_h)$, and $-(\delta+u_1+\mu_h)$. Since all eigenvalues are negative, then condition (A5) is also satisfied. Hence, the proof is complete.

2 Proof of Theorem 4

The existence and uniqueness of the non-negative bounded solution of the state system for each admissible control pair have been established in the previous section. Hence, it remains to verify that the optimal control problem is well posed.

First, the admissible control set $\mathcal{U}$ is non-empty, closed, convex, and bounded in $L^\infty(0,T)^2$. Therefore, every minimizing sequence

$$\{(u_{1n}, u_{2n})\}_{n=1}^{\infty} \subset \mathcal{U}$$

is uniformly bounded. Since the corresponding state variables are also non-negative and bounded on $[0,T]$, there exists a subsequence, still denoted by

$$\{(u_{1n}, u_{2n})\}_{n=1}^{\infty},$$

that converges weakly-* in $L^\infty(0,T)^2$ to some admissible control pair

$$(u_1^*, u_2^*) \in \mathcal{U}.$$

The right-hand side of system (1) is continuously differentiable with respect to the state variables and is affine with respect to the controls u_1 and u_2. Since the state variables associated with admissible controls are uniformly bounded, the corresponding sequence of state solutions is also uniformly bounded and equicontinuous on $[0,T]$. Thus, by the Arzela–Ascoli theorem, there exists a subsequence of state solutions that converges uniformly to a limiting state X^*, which is the solution of system (1) corresponding to the limiting control pair (u_1^*, u_2^*).

Next, the integrand of the objective functional (2) is given by

$$\mathcal{L}(I_1, I_2, u_1, u_2) = \omega_1 I_1 + \omega_2 I_2 + c_1 u_1^2 + c_2 u_2^2.$$

Since $c_1 > 0$ and $c_2 > 0$, the function $\mathcal{L}$ is convex with respect to (u_1, u_2). Moreover, because all state variables are non-negative and $\omega_1, \omega_2 \geq 0$, we have

$$\mathcal{L}(I_1, I_2, u_1, u_2) \geq c_1 u_1^2 + c_2 u_2^2.$$

Therefore, the objective functional is bounded from below and is weakly lower semicontinuous with respect to the controls. Consequently,

$$J(u_1^*, u_2^*, X^*) \leq \liminf_{n\to\infty} J(u_{1n}, u_{2n}, X_n).$$

Since $\{(u_{1n}, u_{2n})\}$ is a minimizing sequence, it follows that

$$J(u_1^*, u_2^*, X^*) = \min_{(u_1,u_2)\in\mathcal{U}} J(u_1, u_2, X).$$

Hence, an optimal control pair exists.
